# Interleukin-6 is critical in the development of pulmonary vascular disease in *Gcn2-*deficient mice

**DOI:** 10.1073/pnas.2531623123

**Published:** 2026-07-06

**Authors:** Max Schwiening, Qingyue Gao, Mark Southwood, Alexi Crosby, Stephen Moore, Jose A. Valer, Niki Veale, Benjamin J. Dunmore, Paul D. Upton, A. A. Roger Thompson, Nicholas W. Morrell, Stefan J. Marciniak, Elaine Soon

**Affiliations:** ^a^ Cambridge Institute for Medical Research, University of Cambridge, Cambridge CB2 0XY, United Kingdom; ^b^ Department of Medicine, University of Cambridge, Cambridge CB2 0QQ, United Kingdom; ^c^ Medical Research Council Toxicology Unit, University of Cambridge, Cambridge CB2 1QR, United Kingdom; ^d^ Division of Clinical Medicine, University of Sheffield, Sheffield S10 2RX, United Kingdom

**Keywords:** pulmonary veno-occlusive disease, GCN2, interleukin-6

## Abstract

Biallelic mutations in EIF2AK4, encoding Eukaryotic Translation Initiation Factor 2α kinase 4 or General Control Nonderepressible 2 (GCN2), cause pulmonary veno-occlusive disease (PVOD), a fatal form of pulmonary hypertension. The mechanisms linking GCN2 deficiency with pulmonary vascular pathology are poorly understood. To investigate this, we developed two mouse models: genetic ablation of *Gcn2*, to mirror *GCN2*-mutation positive PVOD, and a pharmacological model using mitomycin C, a drug which can cause PVOD as an idiosyncratic drug reaction. Both models were phenotyped, and lungs from wild-type and *Gcn2*-deficient mice were analyzed using single-cell RNA sequencing. We show that homozygous loss of *Gcn2* is sufficient to induce mild pulmonary hypertension in mice. Single-cell transcriptomic profiling identified adventitial fibroblasts as the cell population exhibiting the most *Gcn2*-dependent transcriptional changes. Pathway analysis revealed upregulation of inflammatory signaling in *Gcn2^−/−^* adventitial fibroblasts. Consistent with this, we demonstrate a proinflammatory phenotype in *Gcn2^−/−^* mouse fibroblasts and in *Gcn2^−/−^* mice. Using a mitomycin C–induced murine model, genetic deletion of interleukin-6 (*Il6*) rescued the pulmonary vascular phenotype. Furthermore, chronic lipopolysaccharide exposure exaggerated pulmonary hypertension in *Gcn2^−/−^* mice, and *Il6* ablation rescued both baseline and lipopolysaccharide-exacerbated disease. Pharmacological inhibition or genetic ablation of the Integrated Stress Response, which can be driven by GCN2-activation, phenocopies *Gcn2* deficiency. Therefore, we establish a regulatory effect of an intact GCN2-Integrated Stress Response on IL-6 signaling. Together, we show that interleukin-6 is a critical mediator of both *Gcn2* deficiency–associated and mitomycin C–triggered pulmonary vascular disease in mice and highlight IL-6-dependent pathways as potential therapeutic targets.

## Significance

This study examines the effects of General Control Nonderepressible 2 (GCN2) deficiency on the cardiopulmonary system of mice. We show that loss of GCN2 results in mild pulmonary hypertension and exaggerated interleukin-6 (IL-6) responses to LPS. Chronic exposure to LPS in GCN2-deficient mice worsens the pulmonary hypertensive phenotype. Both this and the baseline phenotype are ameliorated by genetic deletion of *Il6.* In an orthogonal model of pulmonary vascular disease induced by mitomycin-C, IL-6 ablation also rescues the pulmonary vascular phenotype. Targeting *Il6*-dependent pathways may be useful in treating this deadly disease.

Pulmonary arterial hypertension (PAH) is an umbrella term describing diseases characterized by an increase in mean pulmonary artery pressures, with a normal left heart (1). Pulmonary veno-occlusive disease (PVOD) is a rare and deadly form of pulmonary arterial hypertension. The histological hallmark of PVOD is the progressive obliteration of small pulmonary veins and venules by fibrous intimal thickening (2). The clinical presentation is one of worsening breathlessness as the increasing pulmonary vascular resistance results in right heart failure and death. Comparatively little is known about the pathophysiology of PVOD and these patients are generally treated empirically with medications licensed for PAH, with particularly poor outcomes. Most patients with PVOD die within 2 to 3 y from diagnosis without lung transplantation (3).

Biallelic mutations in eukaryotic translation initiation factor 2α kinase 4 or *EIF2AK4* (which encodes general control nonderepressible 2, GCN2), were identified as causative in familial forms of PVOD in 2014 (4). *EIF2AK4* mutations were found in all familial cases and 25% of sporadic PVOD. These patients were also younger at diagnosis and had a worse prognosis than patients with idiopathic pulmonary hypertension (5). Many of these mutations result in either near-complete loss of the protein or its function (4, 6, 7). Levels of GCN2 were also shown to be reduced in sporadic PVOD as well as in heritable and idiopathic PAH (8). The mechanism for this is unclear.

GCN2 is a serine/threonine protein kinase, which phosphorylates the α-subunit of the translation initiation factor eIF2 (9). GCN2 is canonically a sensor of amino acid deprivation and ribosomal stress/stalling. In amino acid deficient-conditions, uncharged tRNAs accumulate, which bind directly to the HisRS-like domain and the C-terminal domain of GCN2. It can also be activated by infections, oxidative stress, and hypoxia. Phosphorylation of eIF2α triggers the Integrated Stress Response, which results in the reduction of translation initiation in general and the upregulation of Activating Transcription Factor 4 to either restore homeostasis, or, if overwhelming, to trigger apoptosis (10).

There is now a robust body of evidence linking GCN2 to control of inflammation and immune processes (11, 12). *Gcn2-*deficient mice showed an exaggerated inflammatory response upon exposure to 2% dextran sodium sulfate (DSS), which triggers an inflammatory colitis. This was characterized by Ravindran et al. (11), who proved that *Gcn2^−/−^*mice showed greater weight loss, higher levels of IL-17, and more severe intestinal histological changes post–DSS exposure. Similarly, *Gcn2-*deficient mice developed greater weight loss, higher levels of tumor necrosis factor-α and pancreatitis-associated protein mRNA expression, more severe acinar cell histological changes postexposure to asparaginase (a chemotherapy agent commonly used in acute lymphoblastic leukemia) (12).

Pulmonary arterial hypertension (PAH) in general also has a strong association with inflammatory phenotypes in cell lines, animal models, and patients (13). Idiopathic and heritable PAH patients have higher levels of proinflammatory cytokines, and these levels predict mortality (14, 15). Chronic exposure to proinflammatory insults replicates the PAH phenotype in mice bearing mutations in bone morphogenetic protein receptor type 2, *Bmpr2* (16); the other main genetic cause of PAH. Transgenic rodents overexpressing interleukin-6 (IL-6) are more susceptible to hypoxia-driven pulmonary vasoconstriction, while mice with impairments in IL-6 signaling are protected from this (17–19).

Interleukin-6 is a pleiotropic cytokine which plays a central role in inflammation, acting as both an amplifier and regulator of the immune and inflammatory responses (20). Classical IL-6 signaling occurs when IL-6 binds to a membrane-bound IL-6 receptor α-subunit, which is expressed mainly by hepatocytes and certain leukocytes and is thought to have protective and regulatory effects (21). Trans-signaling occurs when IL-6 complexes with a soluble IL-6 receptor and this activates the membrane signal-transducing receptor glycoprotein 130 (gp130) on target cells. This widens considerably the types of cells which are able to respond to IL-6, as gp130 is ubiquitous; and is thought to be proinflammatory and drive disease pathogenesis. Cluster-signaling is the third form, where complexes of IL-6 and membrane-bound IL-6 receptors on one cell activate gp130 on its neighbors (22). All modes of signaling activate the intracellular Janus kinase signal transducer and activator of transcription (JAK-STAT) pathway, which leads to specific gene regulation. There is increasing evidence that IL-6 driven inflammation is crucial in vascular disease, including other genetic forms of pulmonary hypertension (16), atherosclerosis (23), and heart failure with preserved ejection fraction (24).

Therefore, we hypothesized that *Gcn2* loss leads to a proinflammatory state in the lung, specifically driven by IL-6, which then triggers and propagates the pathogenesis of pulmonary vascular disease. We hypothesize that interruption of the proinflammatory pathways involved would abrogate the pulmonary vascular response.

## Results

### Gcn2 Deficiency in Mice Produces a Mild Pulmonary Hypertensive Phenotype.

**Fig. 1.**
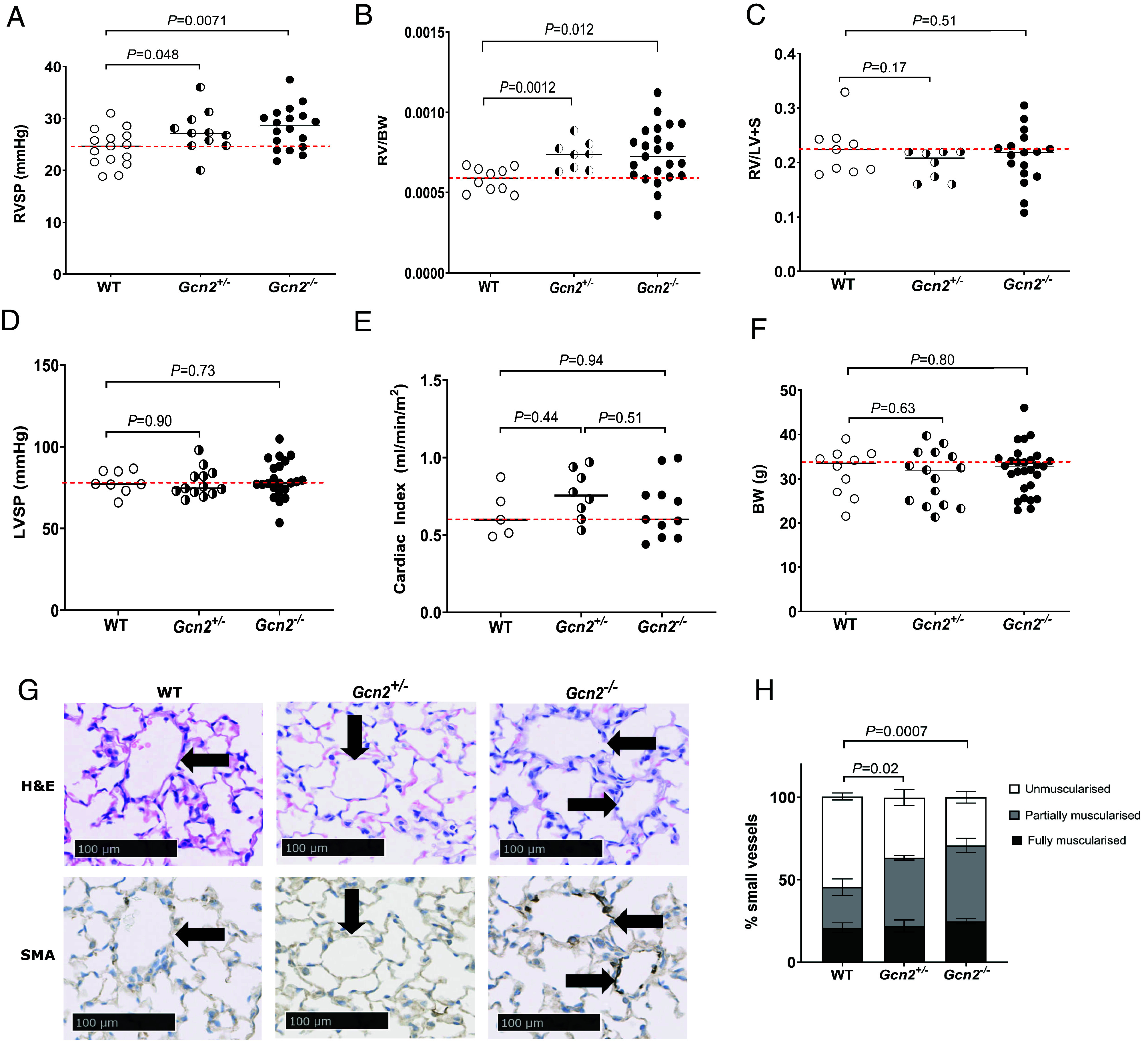
Characterization of the *Gcn2*-deficient mouse. Fig. 1 shows the results of cardiopulmonary phenotyping of wild-type, *Gcn2^+/−^*, and *Gcn2^−/−^* mice. Panels *A*–*C* show the right ventricular systolic pressures (RVSP, panel *A*), right ventricular mass indexed to body weight (RV/BW, panel *B*) and right ventricular mass indexed to septal and left ventricular mass (RV/LV+S, panel *C*) in wild-type, *Gcn2^+/−^*, and *Gcn2^−/−^* mice. Panels *D*–*F* show the left ventricular systolic pressure (LVSP, panel *D*), cardiac index (cardiac output per body surface area, panel *E*), and body weight (panel *F*) of wild-type, *Gcn2^+/−^*, and *Gcn2^−/−^*mice. Panel *G* shows representative lung sections from wild-type, *Gcn2^+/−^*, and *Gcn2^−/−^*mice, stained with hematoxylin and eosin (H&E) and smooth muscle actin (SMA). Black arrows indicate the smaller pulmonary vessels (50 to 250 µm). Panel *H* shows the quantification of nonmuscularized, partially muscularized, and fully muscularized vessels. We note that numbers of animals are not necessarily the same in panels of the same model as not all animals could be used for multiple procedures (right and left heart catheterization, echocardiography, and tissue collection) as the combination(s) are technically challenging. This also applies to Figs. 3–5.

Our first objective was to define the cardio-pulmonary phenotype in *Gcn2-*deficient mice at baseline. We subjected 4.5 to 6-month-old wild-type, *Gcn2^+/−^*, and *Gcn2^−/−^* mice to right and left heart catheterization and echocardiography. We specifically chose this timepoint as certain mutations only manifested their cardiovascular phenotype in aged mice (25). Both *Gcn2^+/−^* and *Gcn2^−/−^* mice displayed increased right ventricular systolic pressures (RVSP) compared to wild-type littermates (24.3 ± 3.5 mmHg in wild-type, versus 27.4 ± 4.1 mmHg in *Gcn2^+/−^* [*P* = 0.048] and 28.2 ± 4.1 mmHg in *Gcn2^−/−^* mice [*P* = 0.0071], Fig. 1*A*). This was associated with a concomitant increase in the right ventricular mass indexed to body weight (RV/BW, Fig. 1*B*). The Fulton index (right ventricular mass indexed to the mass of the left ventricle and septum, RV/LV+S) did not differ between genotypes (Fig. 1*C*). This is due to a concomitant increase in the left ventricular mass indexed to body weight (*SI Appendix*, Fig. S1*A*). The left ventricular systolic pressures (LVSP, Fig. 1*D*) and the cardiac index (cardiac output/body surface area, Fig. 1*E*) did not differ between the genotypes. The left ventricular anterior wall thickness in diastole (LVAW:D, *SI Appendix*, Fig. S1*B*), the left ventricular posterior wall in diastole (LVPW:D, *SI Appendix*, Fig. S1*C*) and the body weight (Fig. 1*F*) also did not differ between genotypes. When the smaller pulmonary vessels (50 to 200 µm) were examined, there was a change in the pattern of vessel muscularization, as shown by the degree of α-smooth muscle actin staining (Fig. 1*G*). Wild-type mice had predominantly unmuscularized vessels, while both *Gcn2^+/−^* and *Gcn2^−/−^*mice had more partially muscularized vessels (Fig. 1*H*). Therefore, there is a subtle pulmonary vascular phenotype in the baseline *Gcn2-*deficient mouse.

### Single-Cell RNA Sequencing Provides Insights Into the Pathways and Cell Types Responsible for the Phenotype Associated with Gcn2 Deficiency.

**Fig. 2.**
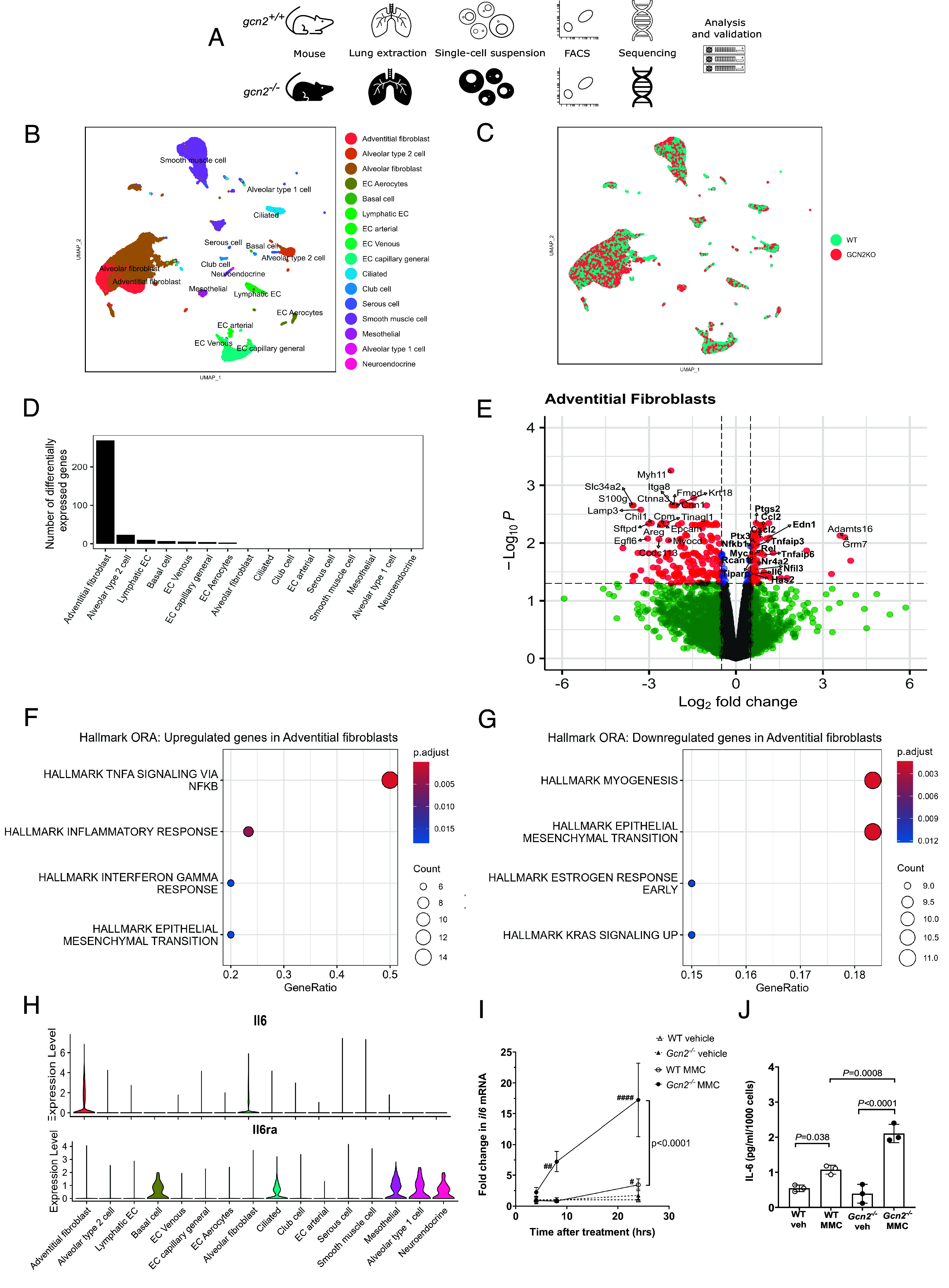
Single-cell RNA sequencing of wild-type and *Gcn2-*deficient mouse lungs. Fig. 2 shows the results of single-cell RNA sequencing of wild-type and *Gcn2^−/−^* mouse lungs. Panel *A* shows an experimental schematic. Panels *B* and *C* show the Uniform Manifold Approximation and Projection graphs (UMAPs) of CD45-negative lung cells, with panel *B* depicting cell clusters and panel *C* showing contributions from the wild-type and *Gcn2^−/−^* mice. Panel *D* shows the cell types from the CD45-negative group ranked by the number of genes differentially expressed between wild-type and *Gcn2^−/−^*. Panel *E* shows a volcano plot of differentially expressed genes between genotypes in adventitial fibroblasts, with genes from upregulated inflammatory pathways identified by ORA in bold. Panels *F* and *G* show pathways that were activated or suppressed in the *Gcn2^−/−^* adventitial fibroblasts with respect to the wild-type. Panel *H* shows violin plots showing the relative expression of *Il6* and *Il6ra* in cell types from the CD45-negative dataset. Panel *I* shows the levels of IL-6 mRNA from wild-type and *Gcn2^−/−^* fibroblasts after exposure to either vehicle or 1 µg/mL of MMC for 4, 8, and 24 h. Panel *J* shows the quantification of secreted IL-6 from wild-type and *Gcn2^−/−^* fibroblasts after exposure to either vehicle or 1 µg/mL of MMC for 24 h.

*Gcn2/GCN2* (*Eif2ak4/EIF2AK4*) is widely expressed in both mouse and human lung [*SI Appendix*, Figs. S2 and S3, data from the mouse lung cell atlas (26) and the integrated Human Lung Cell Atlas v1.0 (27)]. Our next step was to investigate which cells and pathways might be responsible for the pulmonary vascular phenotype. To do this in an unbiased manner, the lungs of *Gcn2*^−/−^ and wild-type littermates were extracted and single-cell suspensions generated for single-cell RNA sequencing (scRNAseq, Fig. 2*A*). Isolation of some cell types, particularly immune cells, is more efficient than others and can skew datasets (28). We therefore sorted cells by CD45 expression to generate CD45-positive (immune cells) and CD45-negative populations (all other cell types, *SI Appendix*, Fig. S4). The Uniform Manifold Approximation and Projection (UMAP) protocol was used for dimensionality reduction and cell clusters from the CD45-negative group (Fig. 2 *B* and *C*) and CD45-positive group (*SI Appendix*, Fig. S5 *A* and *B*) were identified by known marker genes.

A pseudobulk quasi-likelihood model (29) was used to identify genes differentially expressed between wild-type and *Gcn2^−/−^* cells for both the CD45-negative (Fig. 2*D*) and CD45-positive (*SI Appendix*, Fig. S5*C*) populations. Adventitial fibroblasts were the cell type with the highest number of differentially expressed genes by a considerable margin, with 269 differently expressed genes compared to <25 for the second and third ranked cell types. We examined the differentially expressed genes in adventitial fibroblasts in greater detail (volcano plot in Fig. 2*E*). Overrepresentation analysis of differentially expressed genes reveals upregulation of inflammatory signaling pathways (Fig. 2 *F* and *G*). The key inflammatory cytokine detected was *Il6.* We interrogated *Il6* expression in all cell types and found that adventitial fibroblasts demonstrated the greatest expression of *Il6* (Fig. 2*H* and *SI Appendix*, Fig. S5*D*). Expression of its receptor *Il6ra* was more diffuse. A table showing specific genes and the roles of their encoded proteins is shown in *SI Appendix*, Fig. S5*E*.

We therefore hypothesized that GCN2*-*deficient fibroblasts would have a proinflammatory response in vitro. To test this, we exposed wild-type and *Gcn2^−/−^* mouse embryonic fibroblasts (MEFs) to mitomycin C (MMC) in vitro for 4, 8, and 24 h. MMC is a chemotherapeutic agent used in certain cancers and has the idiosyncratic side effect of causing pulmonary veno-occlusive disease which is clinically similar to *GCN2-*mutation associated PVOD (30). *Gcn2^−/−^* MEFS produced significantly greater levels of *Il6* mRNA than the wild-type after MMC exposure at 8 and 24 h (Fig. 2*I*). This was confirmed using ELISAs to measure secreted IL-6 levels at the 24-h time point (Fig. 2*J*). From this we infer that GCN2 loss in fibroblasts leads to greater production of IL-6 after MMC exposure.

### Genetic Ablation of Il6 Is Protective in a Murine Model of Mitomycin C–Induced Pulmonary Hypertension.

**Fig. 3.**
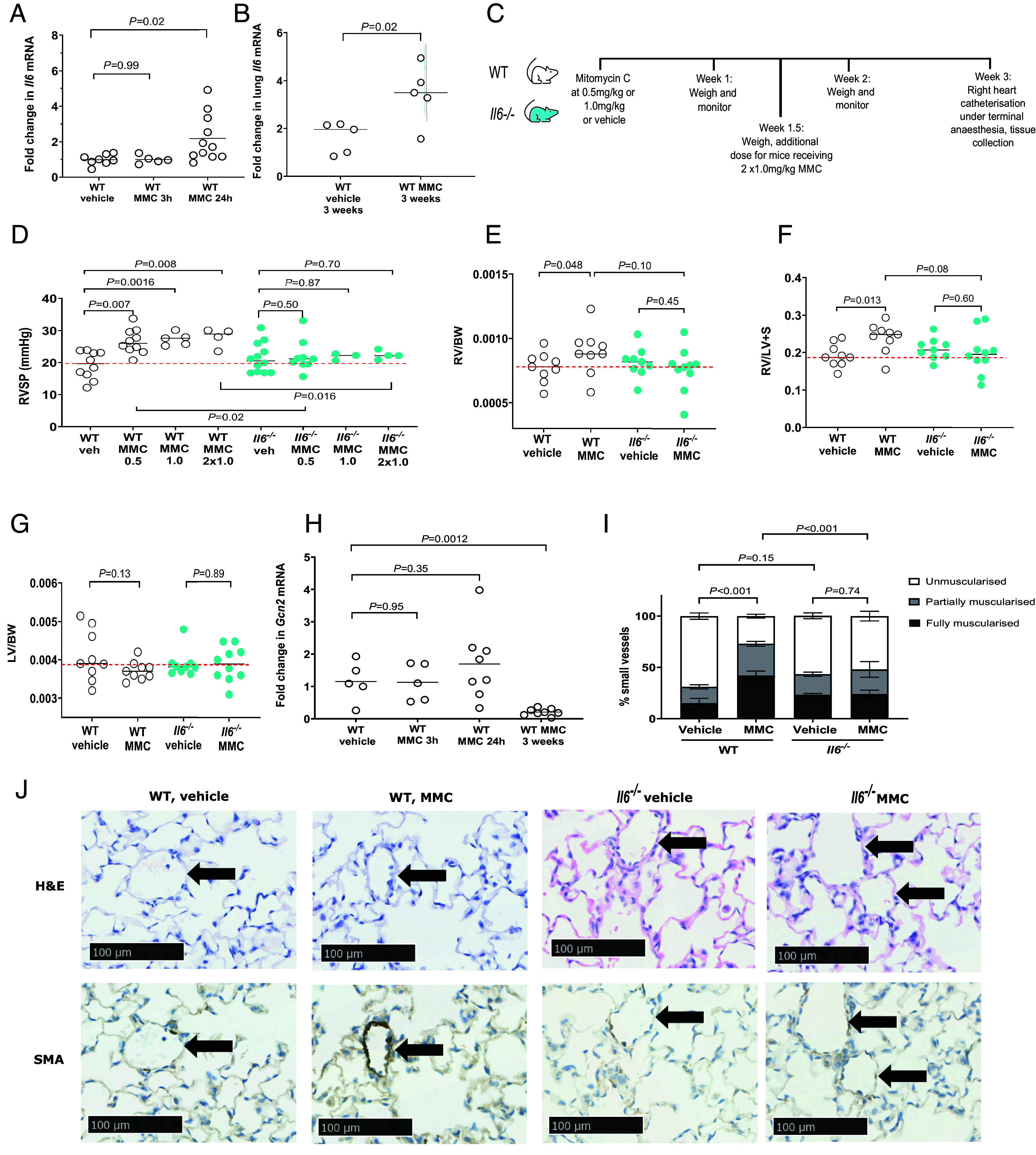
The characterization of a murine model of mitomycin C (MMC) induced PVOD. Fig. 3 shows the characterization of MMC-induced pulmonary vascular disease. Panels *A* and *B* show the levels of IL-6 mRNA in wild-type mouse lungs after exposure to either vehicle or 1.0 mg/kg MMC for either 3 or 24 h (panel *A*), and after 3 wk (panel *B*). Panel *C* shows an experimental schematic in which wild-type and *Il6^−/−^* mice were exposed to either vehicle or 0.5 mg/kg, 1.0 mg/kg, or 2.0 mg/kg (in total) of MMC. Panel *D* shows the right ventricular systolic pressure (RVSP) for all mice. Panels *E*–*G* show the right ventricular mass indexed to body weight (RV/BW, panel *E*), right ventricular mass indexed to left ventricular and septal mass (RV/LV+S, panel *F*) and the left ventricular mass indexed to body weight (LV/BW, panel *G*) for wild-type and *Il6^−/−^* mice exposed to vehicle or 0.5 mg/kg of MMC. Panel *H* shows the levels of lung GCN2 mRNA in wild-type mice after exposure to either vehicle or MMC for 3 h, 24 h, and 3 wk. Panels *I* and *J* show representative lung sections of wild-type and *Il6^−/−^*mice from both vehicle and MMC-treated arms, stained with hematoxylin and eosin (H&E) and smooth muscle actin (SMA); and the quantification of nonmuscularized, partially muscularized and fully muscularized vessels.

We then hypothesized that mitomycin C would induce an IL-6 response in vivo and prolonged exposure could trigger pulmonary vascular disease in mice. To determine if acute exposure to MMC elicited a specific increase in IL-6, 6- to 8-week-old wild-type mice were exposed to 1.0 mg/kg of MMC or vehicle. Lung mRNA encoding IL-6 was increased at 24 h by MMC treatment (Fig. 3*A*) and sustained at 3 wk (Fig. 3*B*). To test the second part of our hypothesis, we developed a mouse model of disease utilizing MMC. Wild-type or *Il6^−/−^* mice aged 8 to 11 wk were injected with cumulative doses of MMC totaling 0.5 mg/kg, 1.0 mg/kg, or 2.0 mg/kg; or vehicle alone, and subjected to right heart catheterization 3 wk later (Fig. 3*C*). Wild-type mice exposed to MMC developed an increase in their right ventricular systolic pressures (Fig. 3*D*), right ventricular mass indexed to body weight (Fig. 3*E*) and right ventricular mass indexed to left ventricular mass (Fulton index, Fig. 3*F*). Strikingly, interleukin-6-deficient mice were protected from the development of elevated RVSP at all doses (Fig. 3*D*). There was no change in the left ventricular mass indexed to body weight (Fig. 3*G*) for both genotypes. Since previous studies have linked MMC exposure with decreased GCN2 expression (7), we interrogated GCN2 mRNA levels in these lungs and found a decrease at the 3-wk point (Fig. 3*H*). Examination of lung sections revealed increased muscularization of the smaller vessels in the MMC-exposed wild-type lungs (Fig. 3 *I* and *J*). These changes did not occur in *Il6^−/−^* mice.

### Gcn2 Deficiency Is Associated with a More Marked Inflammatory Response to Lipopolysaccharide. Chronic Exposure to Lipopolysaccharide Exaggerates the Pulmonary Hypertensive Phenotype in Gcn2-Deficient Mice.

**Fig. 4.**
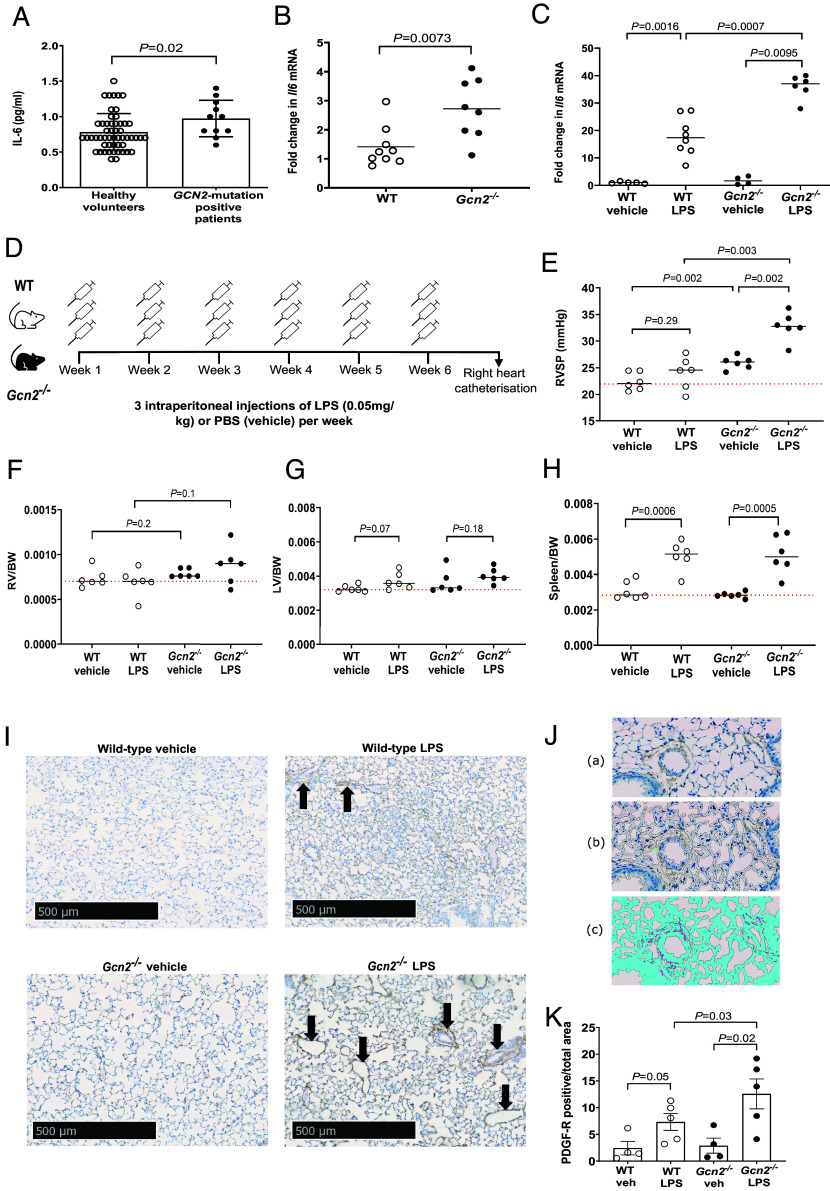
*Gcn2* deficiency is associated with increased IL-6. Chronic LPS exposure worsens pulmonary hypertension in *Gcn2-*deficient mice. Fig. 4 shows the results of IL-6 profiling in GCN2 deficiency and characterization of the acute and chronic response to LPS in *Gcn2-*deficient mice. Panel *A* shows plasma IL-6 levels from healthy volunteers and PVOD patients bearing biallelic GCN2 mutations. Panel *B* shows the levels of IL-6 mRNA from the lungs of baseline aged wild-type and *Gcn2^−/−^*mice. Panel *C* shows the fold change in lung IL-6 mRNA after exposure to either vehicle or 0.05 mg/kg of LPS in young wild-type and *Gcn2^−/−^* mice. Panel *D* shows a schematic for chronic LPS administration. Panel *E* shows the right ventricular systolic pressures (RVSP), panel *F* the right ventricular mass indexed to body weight (RV/BW), panel *G* the left ventricular mass indexed to body weight (LV/BW), and panel *H* the spleen indexed to body weight (spleen/BW) of wild-type and *Gcn2^−/−^* mice, exposed to either LPS or vehicle. Panel *I* shows representative sections of wild-type and *Gcn2^−/−^* mouse lungs, exposed to either LPS or vehicle, stained for PDGF-R. Panel *J* shows the Visiopharm algorithm to quantify PDGF-R staining. Subpanels show the initial histological section (a), the delineation of tissue (dotted outlines, b); and the identification of PDGF-R positivity (pink), and tissue area (turquoise, c). Panel *K* shows the quantification of PDGF-R positivity.

We next sought to substantiate the theory that GCN2 deficiency is associated with a general proinflammatory phenotype. To do this, we obtained plasma samples from GCN2 mutation–bearing patients and healthy volunteers. Our *GCN2* mutation–bearing PVOD patients had higher levels of IL-6 compared to age- and sex-matched healthy volunteers (Fig. 4*A*), while levels of soluble IL6 receptor were unchanged (*SI Appendix*, Fig. S6*A*). Aged (4.5 to 6 mo) *Gcn2^−/−^* mice displayed an increase in lung mRNA encoding IL-6 compared to their wild-type littermates (Fig. 4*B*); and had increased levels of the soluble IL6 receptor (*SI Appendix*, Fig. S6*B*). IL-6 production was further exaggerated in 8 to 10-week-old mice given 0.05 mg/kg of lipopolysaccharide (LPS) and sacrificed 3 h later (Fig. 4*C*). We hypothesized that since an acute exposure to LPS produced greater levels of IL-6 in *Gcn2^−/−^*mice, chronic exposure could exaggerate differences in the cardio-pulmonary phenotype. To test this, 10 to 14-week-old wild-type and *Gcn2^−/−^*mice were injected with either vehicle or LPS thrice-weekly for 6 wk (Fig. 4*D*). Only *Gcn2^−/−^*mice demonstrated a further increase in their right ventricular systolic pressures after chronic LPS exposure (Fig. 4*E*). Neither ventricular mass index changed in either genotype (Fig. 4 *F* and *G*).

All genotypes displayed an LPS-induced increase in spleen weight indexed to body weight (Fig. 4*H*) compared to vehicle-treated mice of the same genotype, proving that a systemic inflammatory response had been elicited. From this we infer that GCN2 loss causes a proinflammatory phenotype at baseline which is further exaggerated by LPS, and that chronic LPS exposure worsens the pulmonary vascular phenotype solely in the *Gcn2^−/−^*mouse.

To investigate the theory that the fibroblast plays an important role, we stained lung slides from the mice in the chronic LPS protocol for PDGF-R [a fibroblast marker (31), Fig. 4*I*]. PDGF-R positivity was then quantified using Visiopharm software. In brief, the algorithm delineated all nonwhite pixels as “tissue” and scored pixels above a threshold intensity for DAB-positivity as positive (Fig. 4*J*). Therefore, an objective score can be calculated for PDGF-R positivity per tissue area. Both wild-type and *Gcn2^−/−^*mice showed an increase in PDGF-R positivity and after exposure to LPS, with the *Gcn2^−/−^* mice showing a greater increase in fibroblast numbers compared to their wild-type littermates (Fig. 4*K*).

### Genetic Ablation of Il6 Abrogates the Pulmonary Hypertensive Phenotype Both at Baseline and after Exposure to Lipopolysaccharide.

We hypothesized that IL-6 is pivotal in the chronic LPS-driven pulmonary vascular phenotype in *Gcn2^−/−^* mice. To test if this is true, we generated mice deficient in both *Gcn2* and *Il6 (Gcn2^−/−^Il6^−/−^*), aged them to 4.5 to 6 mo and subjected them to right heart catheterization (*SI Appendix*, Fig. S7). Concomitant *Il6* loss reverses the increased RVSP associated with *Gcn2-*loss at baseline (*SI Appendix*, Fig. S7*A*). Interestingly, both *Il6^−/−^* and *Gcn2^−/−^Il6^−/−^* mice had increased right and left ventricular mass indexes (RV/BW, *SI Appendix*, Fig. S7*B* and LV/BW, *SI Appendix*, Fig. S7*C*) compared to the wild-type. However, neither the RV/BW nor the LV/BW of these mice were different from the *Gcn2^−/−^*mice. Neither the *Il6^−/−^* nor the *Gcn2^−/−^Il6^−/−^* mouse was significantly lighter than the wild-type (*SI Appendix*, Fig. S7*D*). We note that *Gcn2^−/−^Il6^−/−^* mice were significantly lighter than the *Gcn2^−/−^*mice. Histological examination revealed that the lungs of baseline *Il6^−/−^* and *Gcn2^−/−^Il6*^−/−^ mice resembled those of the wild-type, with predominantly unmuscularized small pulmonary vessels (*SI Appendix*, Fig. S7 *E* and *F*).

**Fig. 5.**
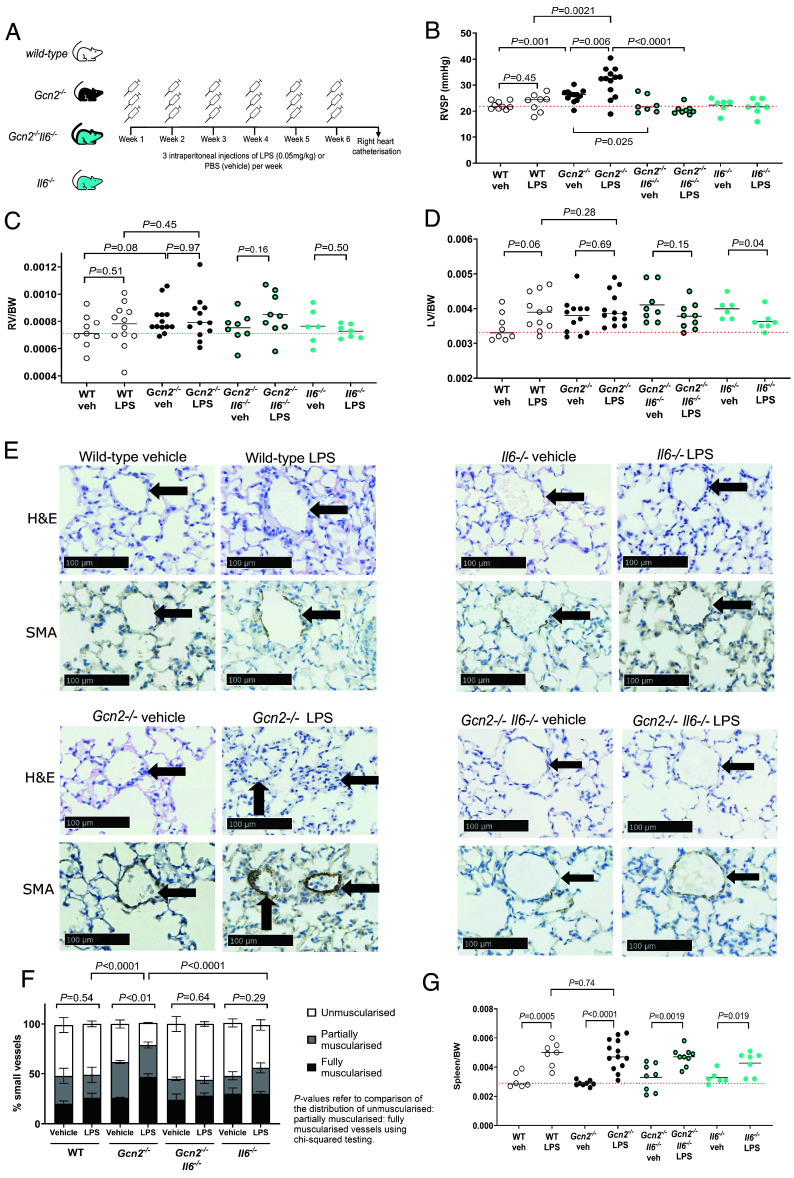
The baseline and exaggerated RVSP response to LPS in *Gcn2-*deficient mice is abrogated by genetic loss of IL-6. Fig. 5 shows the characterization of a murine model of LPS-induced pulmonary vascular disease and reversal of the phenotype by IL-6 ablation. Panel *A* shows the experimental schematic. Panels *B*–*D* show the right ventricular systolic pressure (RVSP, panel *B*), the right ventricular mass indexed to body weight (RV/BW, panel *C*) and the left ventricular mass indexed to body weight (LV/BW, panel *D*). Panels *E* and *F* show representative lung sections of wild-type, *Gcn2^−/−^*,*Il6^−/−^*, and *Gcn2^−/−^Il6^−/−^* mice from the vehicle and LPS-treated arms, stained with hematoxylin and eosin (H&E) and smooth muscle actin (SMA, panel *E*) and the quantification of nonmuscularized, partially muscularized and fully muscularized vessels (panel *F*). Panel *G* shows the spleen weight indexed to body weight for mice from all genotypes.

To test if IL-6 continues to be significant in LPS-induced pulmonary vascular disease, we injected 10 to 14-week-old *Gcn2^−/−^Il6^−/−^* animals, and corresponding wild-type, *Gcn2^−/−^*, and *Il6^−/−^* mice with either LPS or vehicle thrice-weekly for 6 wk (Fig. 5*A*). We have specifically used genetic deletion of IL-6 as being the cleanest possible available model, and also as current commercially available agents such as tocilizumab and sarilumab do not block IL-6 signaling in mice (32). Genetic ablation of *Il6* abrogated the right ventricular systolic pressure increase in both vehicle and LPS-exposed mice (Fig. 5*B*). Neither right nor left ventricular mass index changed appreciably in most genotypes (Fig. 5 *C* and *D*). There was increased muscularization of the smaller lung vessels (50 to 200 μm) as shown by α-smooth muscle actin (SMA) staining in the *Gcn2^−/−^* mice chronically exposed to LPS, which did not occur in the wild-type, *Gcn2^−/−^Il6^−/−^*, or *Il6^−/−^* mice (Fig. 5 *E* and *F*). Splenic enlargement occurred in all genotypes after chronic LPS exposure (Fig. 5*G*), indicating that adequate LPS absorption had occurred to trigger a chronic inflammatory reaction.

**Fig. 6.**
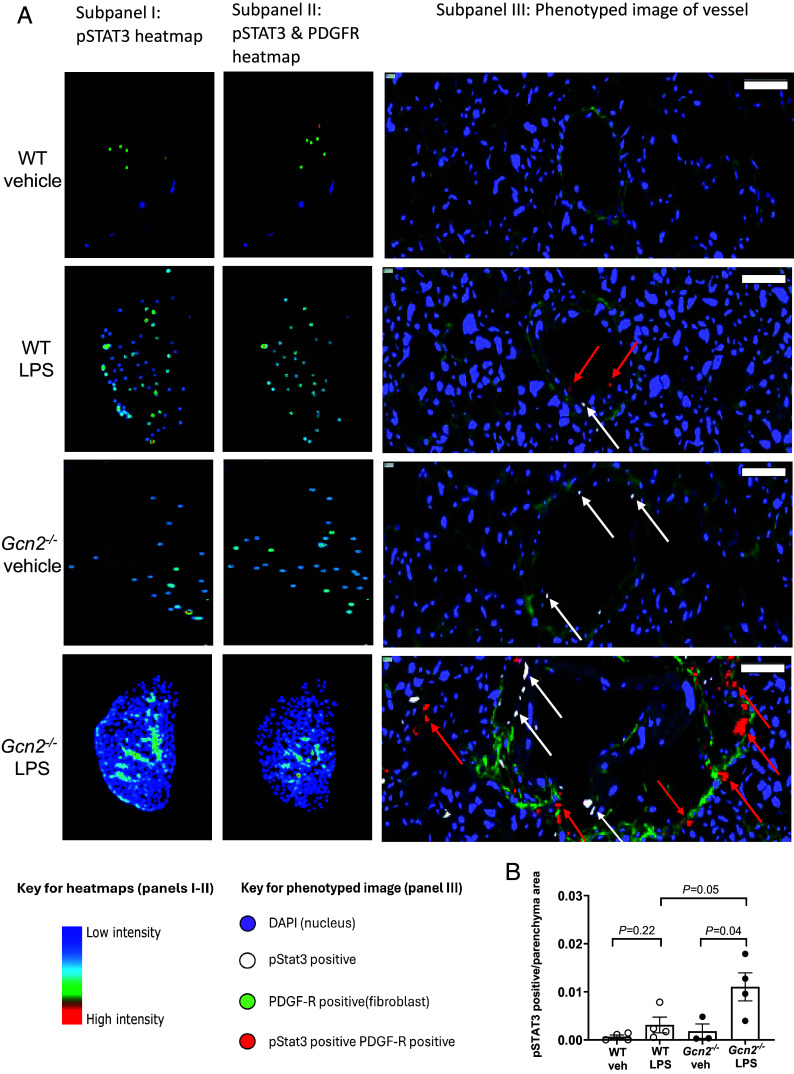
Targets of IL-6 within the mouse lung. Fig. 6 explores the cellular targets of IL-6 in wild-type and *Gcn2^−/−^* mouse lung. Panel *A* shows the results of an immunofluorescence study, where lung sections from wild-type and *Gcn2^−/−^* mice who were exposed to either vehicle or LPS thrice-weekly for 6 wk were stained for pStat3 (marker for IL-6 activation), PDGF-R (a fibroblast marker), and DAPI (to outline nuclei). Subpanel I shows a heatmap of pStat3 staining and subpanel II shows a heatmap showing pStat3 and PDGF-R staining within a mouse lung lobe. Subpanel III shows representative micrographs of a small pulmonary vessel, with lilac nuclear staining, white pStat3 staining, green PDGF-R staining, and red staining for nuclei which are both pStat3-positive and PDGF-R-positive. White arrows demonstrate pStat3-positive cells and red arrows show pStat3-positive and PDGF-R-positive cells. (Scale bars represent 60 µm.) Panel *B* shows quantification for pStat3 staining.

We then sought to identify target cells for IL-6 in this phenotype. To do this, lung sections from wild-type and *Gcn2^−/−^* mice who had been exposed to either 6 wk of LPS or vehicle were stained for phospho-Signal Transducer and Activator of Transcription 3 [pStat3, as a marker for IL-6 response (33)] and PDGF-R [a fibroblast marker (31)]. Subpanel I of Fig. 6*A* shows a heatmap for pStat3, while subpanel II shows the corresponding heatmap for double positive cells (pStat3+PDGF-R+), with a vessel close-up in subpanel III. PStat3 expression is increased in the LPS-exposed *Gcn2^−/−^*lungs (Fig. 6 *A* and *B*) compared to both LPS-exposed wild-type lung and vehicle-exposed *Gcn2^−/−^*lung. In the LPS-exposed *Gcn2^−/−^*lung, there is widespread low-intensity pStat3 staining and medium intensity staining which is predominantly perivascular (Fig. 6*A* subpanel I). This is not seen in the wild-type (either vehicle- or LPS-exposed) or in the vehicle-exposed *Gcn2^−/−^*lung. In the LPS-exposed *Gcn2^−/−^*lungs there are also large numbers of double-positive cells (pStat3+PDGF-R+) in the perivascular space (Fig. 6*A* subpanel III). We infer that fibroblasts are capable of responding to IL-6 in the LPS-exposed *Gcn2^−/−^*lung. There are also pStat3-positive perivascular cells which are PDGF-R negative.

### An Intact GCN2-Integrated Stress Response Axis Restrains the IL-6–pStat3 Response.

**Fig. 7.**
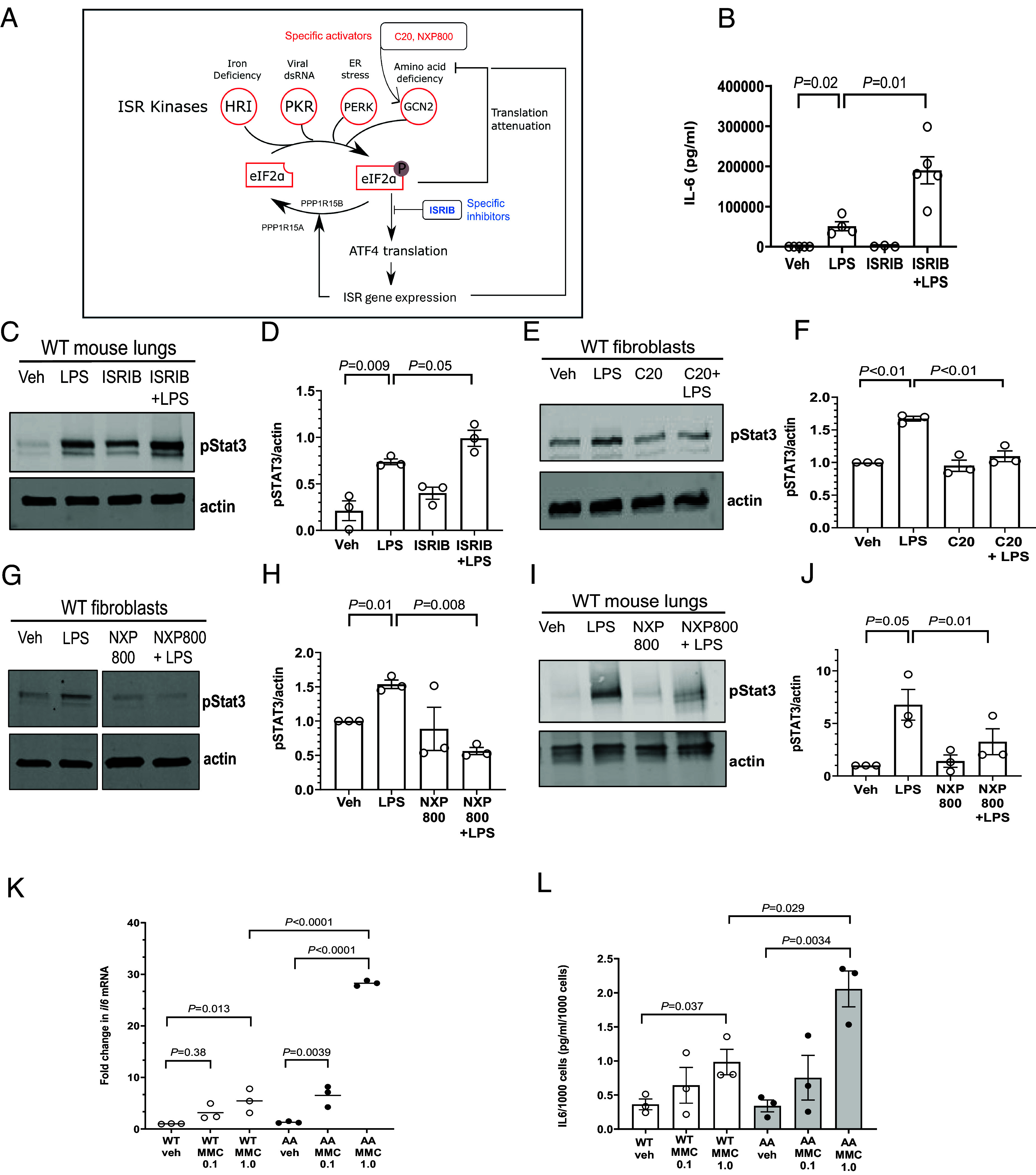
An intact integrated stress response restrains the IL-6–pStat3 axis. Fig. 7 shows the effects of manipulating the Integrated Stress Response on IL-6 and pStat3. Panel *A* shows a schematic for the GCN2-Integrated Stress Response axis. Panel *B* shows IL-6 levels in wild-type mouse sera after intraperitoneal exposure to either vehicle, 0.05 mg/kg of LPS, 5 mg/kg of ISRIB (an ISR-inhibitor) or LPS and ISRIB after 3 h. Panel *C* shows a representative Western blot for pStat3 from wild-type mouse lungs after intraperitoneal exposure to either vehicle, 0.05 mg/kg of LPS, 5 mg/kg of ISRIB or LPS and ISRIB after 3 h; and quantitation of densitometry (panel *D*). Panel *E* shows a representative Western blot for pStat3 from wild-type mouse fibroblasts after treatment with either vehicle, LPS at 10 µg/ml, C20 (a specific GCN2 activator) at 10 µM or C20 and LPS; and quantitation of densitometry (panel *F*). Panel *G* shows a representative Western blot for pStat3 from wild-type mouse fibroblasts after treatment with either vehicle, LPS at 10 µg/ml, 1 µM of NXP800 (a specific GCN2 activator) or NXP800 and LPS; and quantitation of densitometry (panel *H*). Panel *I* shows a representative Western blot for pStat3 from wild-type mouse lungs after intraperitoneal exposure to either vehicle, 0.05 mg/kg of LPS, 20 mg/kg of NXP800 or NXP800 and LPS after 3 h; and quantitation of densitometry (panel *J*). Panels *K* and *L* show the fold change in *Il6* mRNA (panel *K*) and secreted IL-6 (panel *L*) in wild-type and eIF2α^AA^ (AA) fibroblasts after exposure to vehicle, 0.1 µg/mL MMC, or 1.0 µg/mL MMC for 24 h.

Our previous experiments have shown that GCN2 deficiency is associated with an exaggerated IL-6 response (Fig. 4). We sought to explore the interaction between the GCN2-Integrated Stress Response axis and the IL6–Stat3 axis. GCN2 is a serine/threonine protein kinase, which phosphorylates the α-subunit of translation initiation factor eIF2, activating the Integrated Stress Response (ISR; Fig. 7*A*). Pharmacological blockade of the ISR downstream of GCN2 with Integrated Stress Response InhiBitor [ISRIB, (34)] in the presence of LPS creates an exaggerated IL-6 response in wild-type mouse sera (Fig. 7*B*) and an increased phospho-Stat3 (pStat3) response in their lungs (Fig. 7 *C* and *D*). Concomitant administration of a novel GCN2 activator, C20 (35) with LPS, decreased the pStat3 response in wild-type mouse fibroblasts (Fig. 7 *E* and *F*). Another GCN2 activator, NXP800 (36), also significantly ameliorated the pStat3 response in fibroblasts when coadministered with LPS (Fig. 7 *G* and *H*). As NXP800 is orally bioavailable and currently in phase I trials (Clinicaltrials.gov ID 50382), we took this forward to a mouse model. NXP800 also ameliorated the pStat3 response in wild-type mouse lungs when coadministered with LPS (Fig. 7 *H* and *I*).

In an independent genetic model, we used eIF2α^AA^ (AA) fibroblasts, which are unable to mount an ISR due to the mutation of a critical serine at position 51 to alanine (37). These eIF2α^AA^ and corresponding wild-type fibroblasts were exposed to either vehicle or MMC at 0.1 and 1.0 µg/mL for 24 h. The eIF2α^AA^ fibroblasts responded with increased levels of IL-6 mRNA (Fig. 7*M*) and protein (Fig. 7*N*), which phenocopies GCN2 deficiency. Taken together we show that an intact GCN2-integrated stress response axis is capable of restraining the IL6-pStat3 response to both LPS and MMC. Therefore, it is likely that GCN2 deficiency drives an exaggerated IL-6–pStat3 response via a malfunctioning ISR.

## Discussion

We demonstrate that interleukin-6 plays a critical role linking GCN2 loss and the development of a pulmonary vascular phenotype in mice. Our current theory is that GCN2 deficiency causes a dysfunctional integrated stress response, and that this leads to an increased susceptibility to inflammatory insults which manifests as increased IL-6–pStat3 signaling. We show that either pharmacological inhibition of or genetic ablation of the integrated stress response can replicate the exaggerated IL-6 response seen in GCN2 deficiency. The converse is also true, i.e., pharmacological activation of the ISR via GCN2 is capable of ameliorating the IL6-pStat3 response. Ravindran et al. (11) have shown previously that genetic deletion of *Gcn2* in intestinal epithelial cells and antigen-presenting cells increases reactive oxygen species level and this enhances inflammasome activity and IL-1β production. This is a plausible mechanism as we have previously shown that reactive oxygen species are capable of driving IL-6 production and pulmonary vascular remodeling in another genetic model of pulmonary vascular disease (16) and also because the ISR is a key responder in dealing with oxidative stress (38, 39). We think the resultant interleukin-6 driven inflammatory cascade is driven by the adventitial fibroblast. We also show that pulmonary fibroblasts are capable of responding to IL-6 and we hypothesize that this leads to the secretion of promitogenic and proinflammatory factors that promote overgrowth of fibroblasts and smooth muscle cells. Over time this leads to permanent pulmonary vascular remodeling and obliteration of the lumens of the smaller pulmonary vessels, eventually causing pulmonary vascular disease.

There is increasing evidence that this theory is both plausible and consistent. IL-6 is implicated in multiple rodent models of pulmonary hypertension including hypoxia-induced, monocrotaline-induced, and Sugen-5416 and hypoxia models (17–19). Independent of PAH models, IL-6 also mediates adverse cardiac remodeling (40) and a vasoconstrictive profile (41) in rodents. We have shown that IL-6 is elevated in the serum of GCN2-associated PVOD patients. IL-6 levels are an independent risk factor for adverse outcomes in heart failure patients with preserved ejection failure (42, 43). Circulating IL-6 levels have been significantly positively correlated with both pulmonary arterial hypertension and systolic blood pressure elevation (44, 45). On a cellular level, fibroblasts from other diseases, including other pulmonary hypertensive models; also exhibit persistent proinflammatory, proproliferative, and profibrotic phenotypes (46–48), are capable of responding to IL-6 (49) and release promitogenic factors and other proinflammatory cytokines (50).

It is thought that trans-signaling mediates the pathogenic and proinflammatory effects of IL-6 (22). It is interesting to note that our *Gcn2^−/−^*mice had a baseline increase in soluble IL-6 receptor levels when compared to their wild-type littermates, while *GCN2-*mutation–bearing patients do not (*SI Appendix*, Fig. S6 *A* and *B*). We do not yet understand the significance of this finding and it may be related to disease burden—as our mice are do not display overt signs of cardiopulmonary disease; while patients with pulmonary hypertension have a mean time from presentation to diagnosis of greater than 2 y and are usually in symptomatic heart failure at presentation (51).

Loss of GCN2 has been shown in rodents to lead to disruption of T cell development, promotion of proinflammatory cytokine production and to impede recovery from immune and inflammatory insults (11, 52–54). In rats, GCN2 deficiency was strongly associated with a pulmonary IL-6-driven inflammatory response, triggered specifically by acute asparagine and glutamine deprivation (54). We note that the GCN2-deficient rats were so unwell that the experiment was terminated 3 d after injections, which is insufficient time to allow for pulmonary vascular remodeling. This is the reason we have deliberately chosen to test lower doses of MMC and LPS, as we were not seeking to explore acute toxic or septic models but rather to test the premise that low-level inflammation leads to pulmonary vascular remodeling in the context of GCN2 deficiency.

The literature also supports the premise that GCN2 deficiency leads to a more severe inflammatory phenotype in the context of experimental inflammatory colitis (11) and autoimmune neuroinflammation (55). These papers used the same *Gcn2^−/−^* mouse line as this manuscript [B6.129S6-Eif2ak4tm1.2Dron/J (56)]. However, the proinflammatory phenotype appears common to *Gcn2* deficiency, as mice with a missense mutation in *Gcn2* (*atchoum,* which results in a near-complete loss of the protein) have defective responses to cytomegalovirus infection (57), and *Eif2ak4*^K1488X/K1488X^ mice (58) had substantial infiltrates around pulmonary veins.

It is fascinating that the only significant phenotype of *GCN2* mutations in patients is pulmonary veno-occlusive disease. This ties in with our single-cell RNA sequencing results, which indicate that adventitial fibroblasts are a cell type of interest. We note that fibroblast numbers are increased after LPS exposure, and this increase in greater in *Gcn2^−/−^* mouse lungs, providing a targetable source for IL-6. Our work demonstrates that not only can fibroblasts drive production of IL-6, but are also capable of an IL-6 response. Adventitial fibroblasts have recently been found to be critical drivers of vascular remodeling in PAH (47, 48). They are also intimately involved in vascular wall inflammation (59, 60) and so may form the link between GCN2 deficiency, inflammation and vascular remodeling.

This work opens up potential ways of treating *GCN2-*mutation associated pulmonary vascular disease. We hypothesize that either blocking IL-6 or its associated pathways may be helpful; or augmenting the ISR. The first approach has the advantage of being straightforward but runs the risk of increasing the likelihood of infections (61). From our point of view, attractive next steps include trials of new agents such as olamkicept [soluble gp130Fc, (62)], or STAT3 inhibitors such as napabucasin (34); which selectively target IL-6 trans-signaling (for the former) and STAT3 activity (for the latter), prior to translational work.

The second approach, which aims to restore balance by modulating the integrated stress response, is intriguing as it may avoid the immunosuppressive effects of anti-IL-6 approaches and would likely temper other mechanisms through which GCN2 deficiency affects the pulmonary vascular tree and myocardium. Both Zhu et al. (63) and ourselves have shown that *Edn1,* encoding endothelin-1, a potent vasoconstrictor, is affected by the presence of GCN2. Prabhakar et al. (64) used a rat model of MMC to demonstrate that the resultant pulmonary vascular disease was dependent on activation of protein kinase R and ISR, which disrupted the endothelial barrier. From these studies we can surmise that it is highly likely that the GCN2–ISR axis exerts multiple effects through inflammatory, vasoactive, oxidative, and metabolic pathways in the pulmonary vascular tree and that modulating the ISR is a novel therapeutic approach.

To summarize, GCN2 deficiency in mice causes an exaggerated proinflammatory response to lipopolysaccharide and MMC, resulting in pulmonary vascular remodeling and raised pulmonary vascular pressures. IL-6 is critical in this process as genetic ablation of *Il6* abrogates this response. Adventitial fibroblasts may be a key cell type in both producing and responding to IL-6. Taken together we hypothesize that IL-6-dependent pathways and the integrated stress response may be attractive pharmacological targets in *Gcn2-*mutation associated disease.

## Materials and Methods

*Gcn2^−/−^* mice (B6.129S6-Eif2ak4tm1.2Dron/J, https://www.jax.org/strain/ 008240), *Il6^−/−^* mice (B6.129S2-Il6tm1Kopf/J, https://www.jax.org/strain/002650), and double-deficient crosses (*Gcn2^−/−^Il6^−/−^*) were used. Animal studies were conducted in accordance with the UK Animals (Scientific Procedures) Act 1986 under the Home Office project licenses 70/8550 (N.W.M.), 7550697 (N.W.M.), and 5789170 (S.J.M.). The patient study was approved by the National Research Ethics Service East of England committee (references 13/EE/0325 and 13/EE/0203), and all participants gave written informed consent.

Further detailed protocols are available in the *SI Appendix*.

## Supplementary Material

Appendix 01 (PDF)

## Data Availability

The single-cell RNA sequencing data are accessible at the Gene Expression Omnibus database with the reference GSE294702 (65), and detailed methods are provided in the *SI Appendix*.
