# Supplementary material for: Interleukin-6 is critical in the development of pulmonary vascular disease in Gcn2-deficient mice
Source: Proc Natl Acad Sci U S A. 2026 Jul 6;123(28):e2531623123. doi: 10.1073/pnas.2531623123 (PMC13367861; doi:10.1073/pnas.2531623123)
Supplement: Supplementary file 1 — Appendix 01 (PDF) [file pnas.2531623123.sapp.pdf]

Interleukin-6 is critical in the development of pulmonary vascular disease in *Gcn2*-deficient mice: Supplementary Material

Authors: Max Schwiening[1], Qingyue Gao[1], Mark Southwood[2,3], Alexi Crosby[2], Stephen Moore[2], Jose A. Valer[1], Niki Veale[1], Benjamin J. Dunmore[1,2], Paul D. Upton[2], A.A. Roger Thompson[4], Nicholas W. Morrell[2], Stefan J. Marciniak[1], Elaine Soon*[1,2].

Author Affiliations:

[1]Cambridge Institute for Medical Research, University of Cambridge; Cambridge CB2 0XY, UK.

[2]Department of Medicine, University of Cambridge; Cambridge CB2 0QQ, UK.

[3]MRC Toxicology Unit, University of Cambridge; Cambridge CB2 1QR, UK.

[4]Department of Infection, Immunity and Cardiovascular Disease, University of Sheffield; Beech Hill Road, Sheffield S10 2RX, UK.

Corresponding Author Information:

Dr Elaine Soon

Cambridge Institute for Medical Research, Keith Peters Building, Hills Rd, Cambridge CB2 0XY.

Eels2@cam.ac.uk

ORCID ID: 0000-0002-5744-5014

SUPPLEMENT

Supplementary Methods

Quantitative PCR

Mouse embryonic fibroblasts (MEFs) derived from *Gcn2*$^{-/-}$ or wild-type littermate embryos were plated in 6-well plates for RNA studies at densities of 3 x10^5 cells/well. After treatment, cells were lysed and RNA extracted using the RNeasy Mini Kit (Qiagen). The subsequent steps were performed in exactly the same way as tissue RNA. The primers used are shown below in Supplementary Table 1.

**Gene**	**Species**	**Forward 5'-3'**	**Reverse 5'-3'**	**Source**
Actin	Mus musculus	TCCTGGCCTCACTGTCCA	GTCCGCCTAGAAGCACTTGC	W'out *et al*
Chop	Mus musculus	GGAGCTGGAAGCCTGGTATGAG	GCAGGGTCAAGAGTAGTGAAGG	W'out *et al*
Il6	Mus musculus	Qiagen-proprietary Mm_IL6_1_SG	Qiagen-proprietary	
Cxcl1	Mus musculus	Qiagen-proprietary Mm_Cxcl1_1_SG	Qiagen-proprietary	
Eif2ak4	Mus musculus	Qiagen-proprietary Mm_Eif2AK4_1_SG	Qiagen-proprietary	

Supplementary Table 1: Primers used for quantitative PCR

ELISA

For cell experiments, mouse embryonic fibroblasts (MEFs) were plated in 24-well plates for ELISA studies at densities of 3 x10^4 cells/well. After exposure to either the treatment or to vehicle only the supernatants were harvested and the cells in the wells counted for normalization purposes. Flat-bottomed medium-binding 96-well plates (Nunc-Immuno, M9140, Sigma-Aldrich, UK) were coated with 50 microlitre of capture antibody (Supplementary Table 2) diluted in a carbonate buffer for 2 hours at room temperature. The

plate was then washed thrice using PBS containing 0.05% Tween (v/v, PBS-T) and blocked using 5% foetal bovine serum in PBS-T for an hour. 50microlitres of samples and standards were added and the plate incubated overnight at 4oC. After three washes with PBS-T, a biotinylated capture antibody was added and left to incubate for 2 hours. The plate was then washed three times as before and incubated with ExtraAvidin alkaline phosphatase conjugate (Sigma-Aldrich, UK) at 1/400 for a further 2 hours. After a further 2 washes with PBS-T and a final wash with distilled water, the substrate P-nitrophenylphosphate (PNPP, Sigma-Aldrich, UK) was added at a concentration of 1 microgram/ml in diethanolamine buffer (10mM diethanolamine, 0.5mM MgCl2). The absorption at 405nm was measured using a Tecan CM Spark plate reader (Tecan, Switzerland). A four-parameter logistic curve was fitted to the standards and used to interpolate the concentration of the unknown samples. Protein levels were then normalised to the number of live cells in the corresponding well.

**Antibody**	**Species recognised**	**Manufacturer**	**Concentration of antibody**	**Vehicle**
IL-6	Mouse	R&D Systems MAB406	2ug/ml	Carbonate coating buffer
IL-6 biotinylated	Mouse	R&D Systems BAF406	0.3µg/ml	5% foetal bovine serum in PBS-Tween

Supplementary Table 2: Antibodies used for ELISA

Immunohistochemistry

After the lung slides had been stained, they were scanned using a Hamamatsu NanoZoomer XR and analysed using NDP.view2 software (https://www.hamamatsu.com/eu/en/product/life-science-and-medical-systems/digital-slide-scanner/U12388-01.html, Hamamatsu Photonics, Japan). They were then classified by one of three blinded reviewers as being non-muscularised, partially muscularised or fully muscularised, dependent on the degree of smooth muscle-actin staining (Supplementary Table 3).

**Category**	**Degree of muscularisation**	**Representative example**
Non-muscularised	≤10% smooth-muscle actin staining	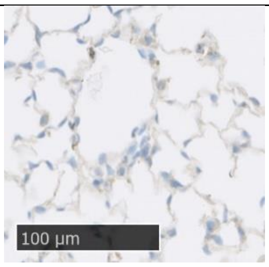
Partially muscularised	>10-90% smooth-muscle actin staining	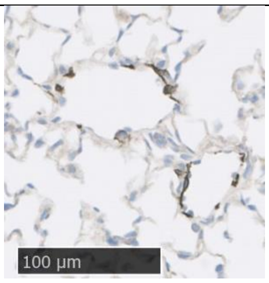
Fully muscularised	>90% smooth-muscle actin staining	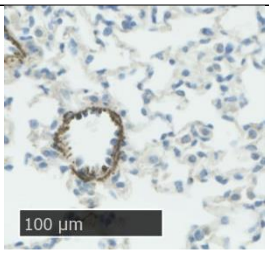

Supplementary Table 3: Definitions used for pulmonary vessel grading

Immunofluorescence

Initial immunohistochemical staining was performed using multiplex immunofluorescence in a single batch using a Ventana Discovery Ultra staining platform (Roche, UK). Rabbit monoclonal anti-PDGFR (Abcam, ab203491, 1:800) and rabbit monoclonal pSTAT3 (Cell Signalling Technology, #9145, 1:50) were stained sequentially and labelled with Omni-map anti-rabbit secondary anti-sera (Roche, UK), visualised using Opal520 and Opal690 respectively (Akoya Bioscience, USA) and counterstained with DAPI (Roche, UK). All immunostained slides were scanned using a Vectra3 multispectral quantitative imaging system (Akoya Biosciences, USA) with identical exposure times used for each slide and using DAPI as the overview filter. Image analysis was performed using Visiopharm 2026.02.2x64. Briefly, a tissue identification app using DAPI thresholding was used to identify all tissue and a second app used to reclassify and measure parenchymal and airspace area. A third app was developed based on the Visiopharm 'quick-start' nuclear detection to 1) identify all nuclei (lilac objects), then modified using multiple 'change by intensity steps' to reclassify nuclei as 2) pSTAT3-positive (based on Opal690 pixel intensity, white objects) and 3) to then change pSTAT3-positive nuclei (white objects) to pSTAT3-PDGFR dual positive (red objects) based on Opal520 pixel intensity. A counting frame was applied and images were batch processed. Cell counts for each phenotype were normalised to parenchymal area.

Western blotting

Flash-frozen mouse lungs were homogenised in radioimmunoprecipitation assay buffer (IgEpal, 10% SDS, sodium deoxycholate and distilled water) containing an EDTA-free protease inhibitor (Roche, Indianapolis, IN) at 4°C using sterile 3mm steel beads in a tissue

lyser (PowerLyzer, Qiagen). The protein lysates were then centrifuged at 10000g for 10 minutes to remove any suspended matter. Mouse fibroblasts were snap frozen in the same RIPA buffer and gently scraped to generate protein lysates. These were fractionated on sodium dodecyl sulfate–polyacrylamide gel electrophoresis gels and immunoblotted. Blots were blocked with 5% nonfat milk and incubated overnight at 4°C with primary antibodies (see the online supplement). Blots were reprobed with an anti-actin antibody to provide loading controls.

**Antibody**	**Species recognised**	**Manufacturer**	**Concentration of antibody**	**Vehicle**
Phospho-Stat3	Mouse	Cell Signalling Technology	1:1000	5% foetal bovine serum in TBS-Tween
Actin	Mouse	R&D Systems	1:1000-1:2000	5% foetal bovine serum in TBS-Tween

Supplementary Table 4: Antibodies used for Western blotting

Patient genotyping

All patients had next-generation paired-end whole genome sequencing using Illumina HiSeq2500 and HiSeqX (Illumina, San Diego, USA). DNA was extracted from whole blood at the central extraction and QC laboratory in Cambridge. Reads were aligned against the

Genome Reference Consortium human genome build 37 (GRCh37, GenBank:2648) using the Illumina Isaac Aligner (version SAAC00776.15.01.27) and variants were then called using the Illumina Starling Software (version 2.1.4.2)[1]. The variants were then left-aligned, normalised with BCFtools and loaded into our Hbase database (Wilmington, USA) to produce multi-sample variant calls for the genetic association studies[2].

Cytokine measurements in patient plasma

A custom multiplex assay manufactured by Meso Scale Discovery (MSD, Maryland, USA) was used to measure cytokine and growth factor levels in patient plasma. Calibration curves were prepared in the assay diluent, with a range of 1.2 to 40,000pg/ml, dependent on the cytokine. Arrays were pre-incubated with 25 microlitres per well of assay diluent for 30 minutes. 25 microlitres of sample or calibrator were added in duplicate to wells in the plate and then incubated at room temperature for 2 hours. The array was washed with PBS-Tween, and 25 microlitres of detection antibody was added. After a further 2 hours of incubation at room temperature, the array was washed and the detection solution added. Results were read using an MSD Sector Imager 6000 (Maryland, USA). Cytokine concentrations were determined with SoftMax Pro (version 4.6, Molecular Devices, California, USA) using curve fit models.

Single cell RNA Sequencing

A pilot experiment was initially conducted to optimise the protocol for harvesting mouse lungs and dissociating them into suspensions containing only live single cells representative

of the lungs from which they were sourced. This revealed that >90% of cells isolated were CD45-positive. Therefore an additional step was introduced to increase the proportion of non-immune cells so they could be characterised, particularly as endothelial cells and smooth muscle cells are usually thought to be the main cell types of interest in pulmonary hypertension[3, 4]. The single cell suspension was subjected to flow cytometry analysis and sorted based on the haematopoietic marker CD45 (Alexa Fluor® 488 anti-mouse CD45 Antibody, BioLegend, UK) to separate immune and non-immune cells and ensure that the same numbers of each were submitted to allow for single cell RNA sequencing of both compartments (Supplementary Figure 1).

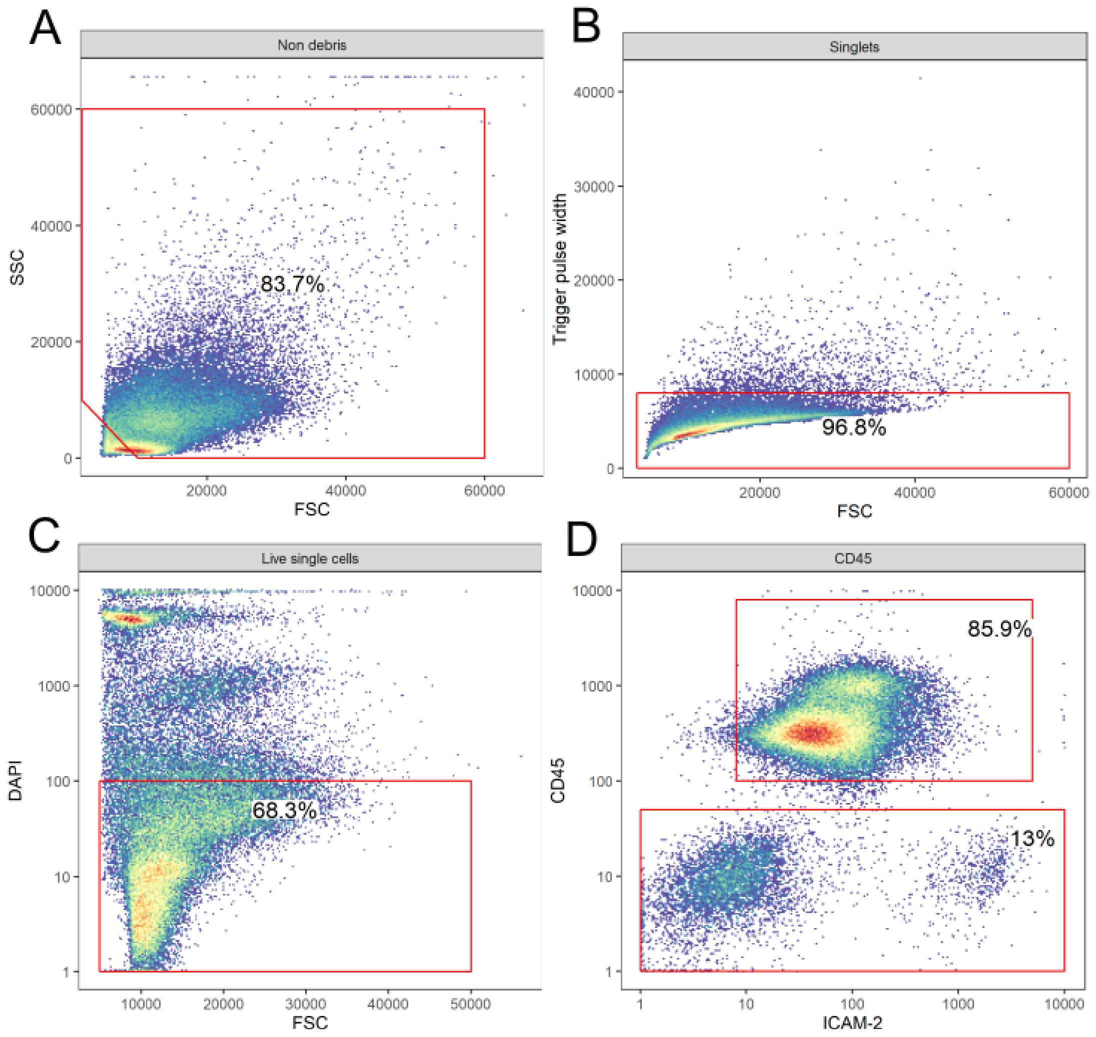

Supplementary Methods Fig. 1: Flow cytometry analysis and sorting for single cell RNA sequencing samples.

Panels A-C show gating used to eliminate debris (panel A), to isolate single cells (panel B), and to sort for live (*i.e.* DAPI-negative) cells (panel C). Panel D shows gating for CD45-positive (blue) and CD45-negative (pink) cells, with additional ICAM-1 staining to visualise the endothelial cell population.

Supplementary Results

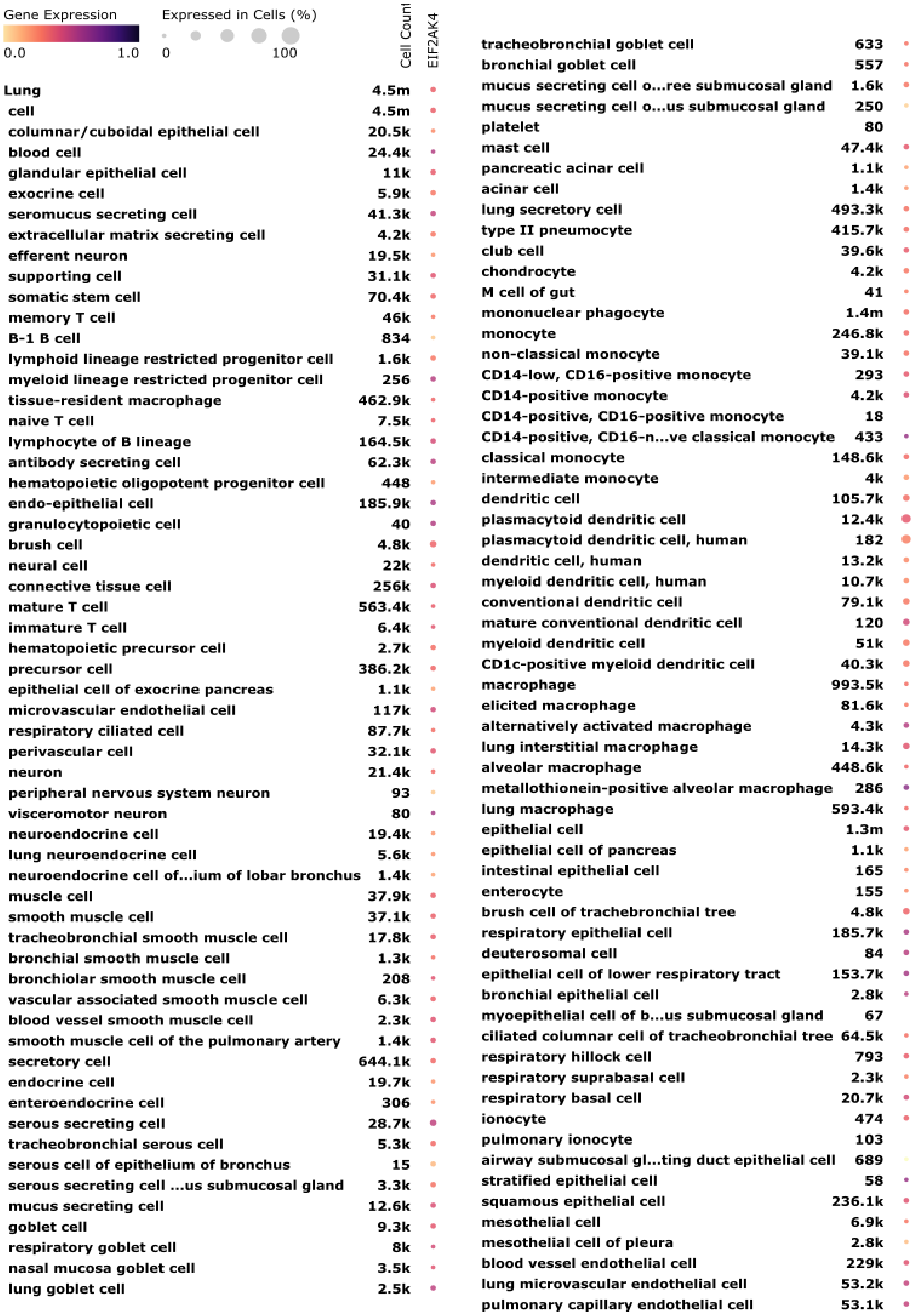

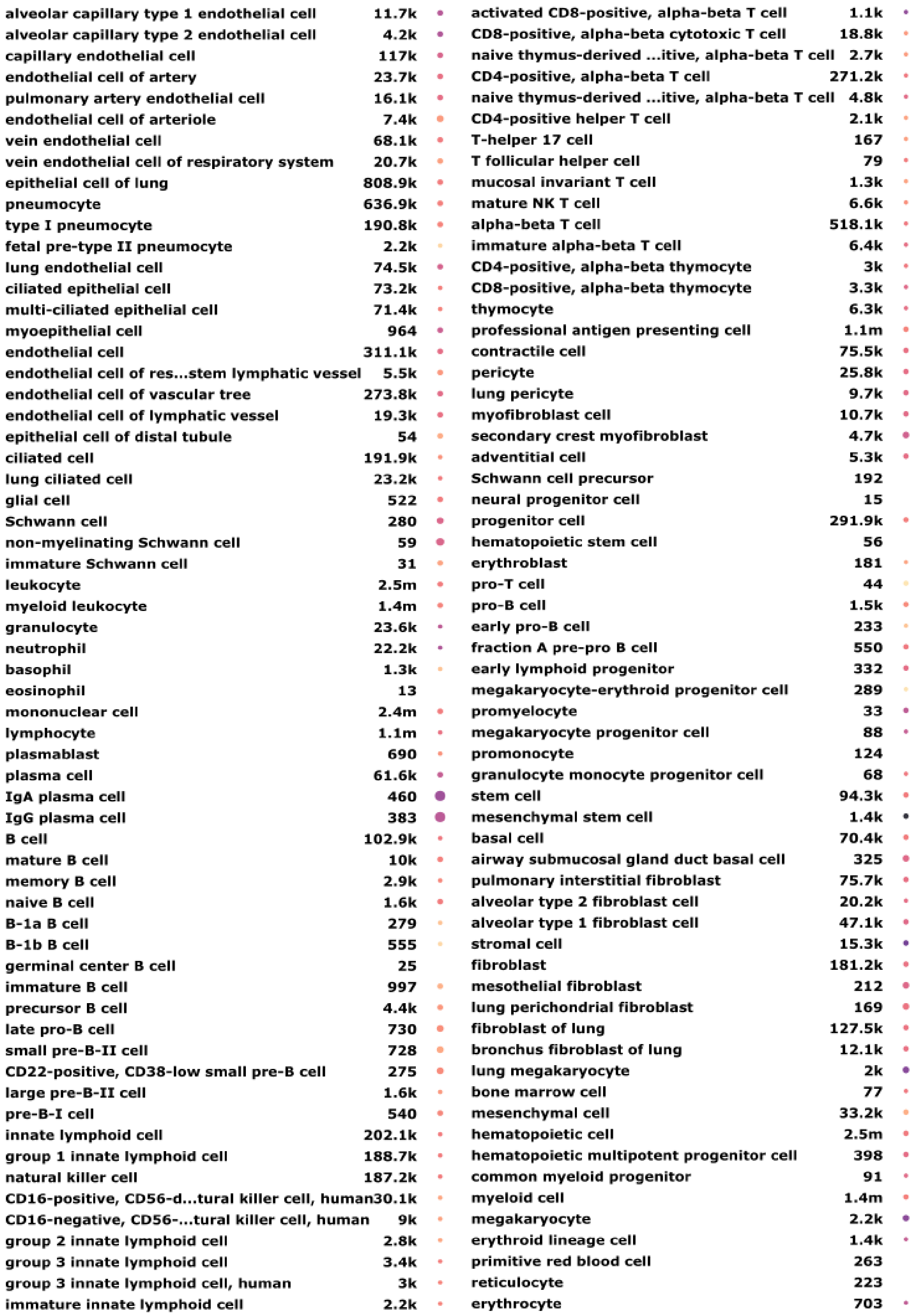

Supplementary Figure 1: Expression of *EIF2AK4* (*GCN2*) in human lung.

Data extracted from the integrated Human Lung Cell Atlas v1.0

(https://data.humancellatlas.org/hca-bio-networks/lung/atlases/lung-v1-0)

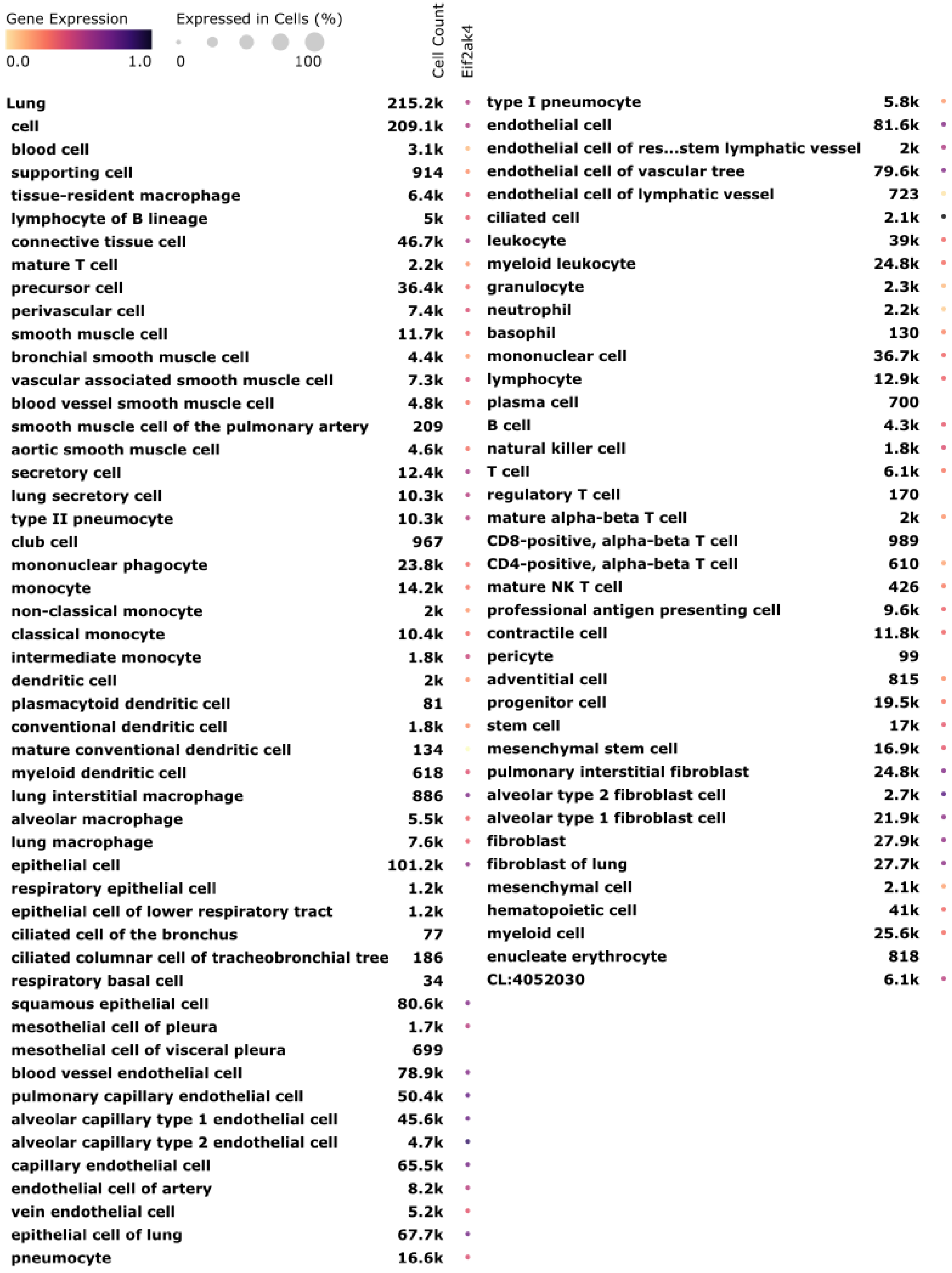

Supplementary Figure 2: Expression of *Eif2ak4* (*Gcn2*) in mouse lung.

Data extracted from the mouse lung cell atlas (https://cellxgene.cziscience.com/gene-expression).

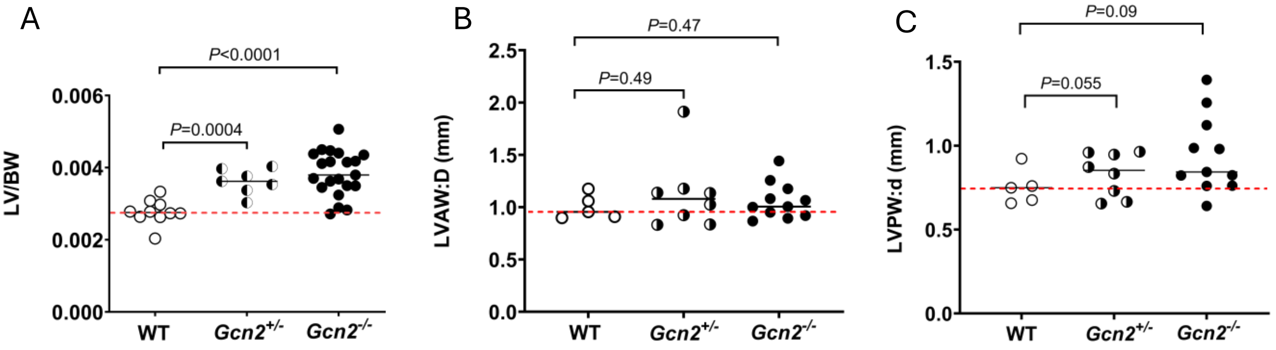

Supplementary Figure 3: Further characterisation of the *Gcn2$^{-/-}$* mouse

Panel A shows the left ventricular mass indexed to body weight (LV/BW) of wild-type, *Gcn2$^{+/-}$* and *Gcn2$^{-/-}$* mice. Panels B-C show the left ventricle anterior wall thickness measured at diastole (LVAW:D, panel B) and the left ventricle posterior wall thickness measured at diastole (LVPW:D, panel C) of wild-type, *Gcn2$^{+/-}$* and *Gcn2$^{-/-}$* mice measured by echocardiography.

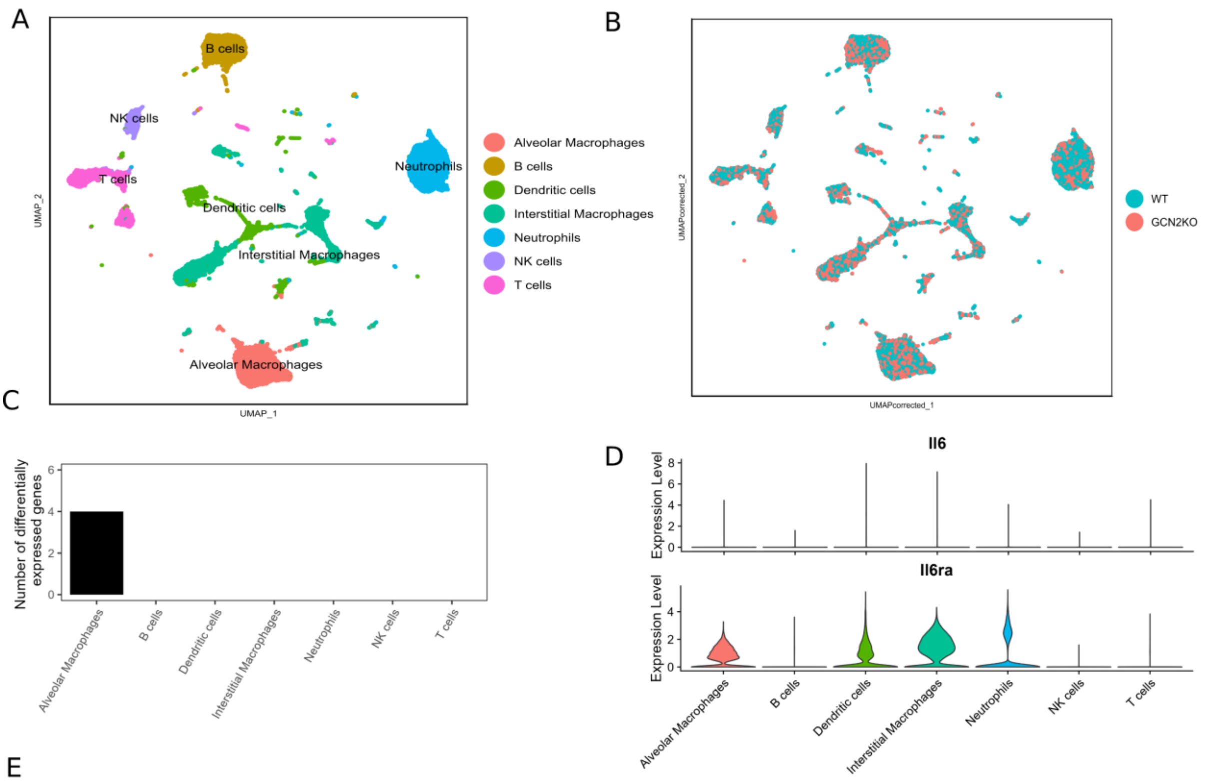

E

Gene	Upregulated in *gcn2*$^{-/-}$ *versus* wild-type adventitial fibroblasts
Adamts16	ADAM metallopeptidase with roles in inflammation, vascular biology and blood pressure control.
Edn1	Endothelin 1, potent vasoconstrictor, endothelin receptor antagonists are used to treat PAH.
Ptgs2	Inducible prostaglandin-endoperoxide synthase, with a particular role in inflammation, activating macrophages and leucocytes.
Ccl2	C-C Motif Chemokine Ligand 2, chemokine involved in in inflammatory and immunoregulatory processes, chemoattractant for monocytes and basophils.
Cxcl2	C-X-C Motif Chemokine Ligand 2, a haemoregulatory chemokine, produced by activated monocytes and neutrophils and expressed at sites of inflammation.
Ptx3	Pentraxin related gene, allows (1->3)-beta-D-glucan binding activity, regulates the nitric oxide biosynthetic process, modulates inflammatory response, associated with right ventricle hypertrophy and implicated as biomarker in pulmonary hypertension.
Tnfaip3	TNF Alpha Induced Protein 3, ubiquitin-editing enzyme, involved in immune and inflammatory responses signaled by cytokines
Tnfaip6	TNF Alpha Induced Protein 6, induced by proinflammatory cytokines, involved in extracellular matrix stability and cell migration.
Nr4a2	Member of the steroid-thyroid hormone-retinoid receptor, important for the differentiation and existence of dopaminergic neurones
Nfil3	Nuclear Factor, Interleukin 3 Regulated, regulates circadian rhythm.
Il6	Interleukin-6, inflammatory cytokine, endogenous pyrogen, implicated in inflammatory and autoimmune diseases.
Has2	Hyaluronan, constituent of the extracellular matrix, actively produced during wound healing and tissue repair.
Grm7	Glutamate receptor, metabotropic 7. Enables calcium channel regulation and voltage gated calcium channel activity.
**Gene**	**Downregulated in *gcn2*$^{-/-}$ *versus* wild-type adventitial fibroblasts**
Myh11	Myosin. Component of muscle myosin complex, enables cytoskeletal and smooth muscle activity.
Itga8	Integrin alpha 8. Generates alpha subunit of the integrin receptor, which mediate cellular interactions with matrix and other cells.
Slc34a2	pH-sensitive sodium-dependent phosphate transporter.
Lamp3	Lysosomal-associated membrane protein 3. Negative regulator of proteolysis, involved in response to interferon-alpha.
S100g	S100 calcium binding protein G. Enables calcium-dependent protein binding.

Supplementary Figure 4: Further interrogation of single-cell RNA sequencing datasets

Panels A and B show the Uniform Manifold Approximation and Projection graphs (UMAPs) of CD45-positive lung cells, with panel A depicting cell clusters and panel B showing contributions from the wild-type and *Gcn2*$^{-/-}$ mice. Panel C shows the cell types from the CD45-negative group arranged in order by the number of genes differentially expressed between wild-type and *Gcn2*$^{-/-}$ lung cells. Panel D shows violin plots showing the relative expression of *Il6* and *Il6ra* in cell types from the CD45-positive dataset. Panel E shows the genes most upregulated and downregulated in *gcn2*$^{-/-}$ versus wild-type adventitial fibroblasts and a summary of their roles. Genes encoding proteins which have inflammatory roles are in red while genes encoding proteins with known roles in pulmonary hypertension, systemic hypertension or vascular biology are in purple.

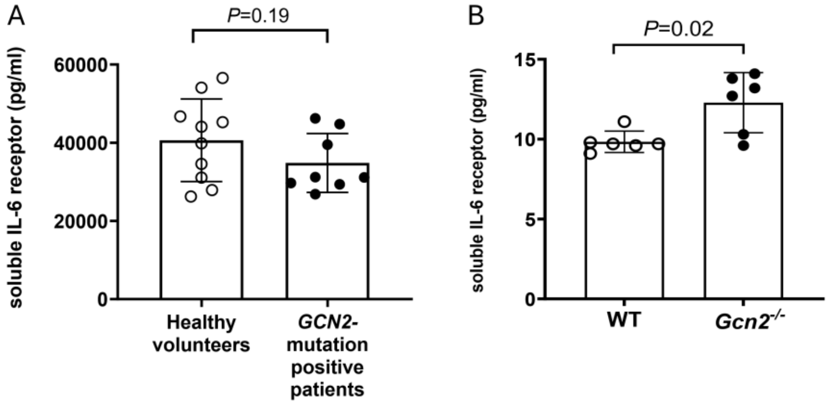

Supplementary Figure 5

Panels A and B show levels of soluble IL-6 receptor from healthy volunteers and GCN2-mutation positive patients (panel A) and wild-type and *Gcn2*$^{-/-}$ mice (panel B).

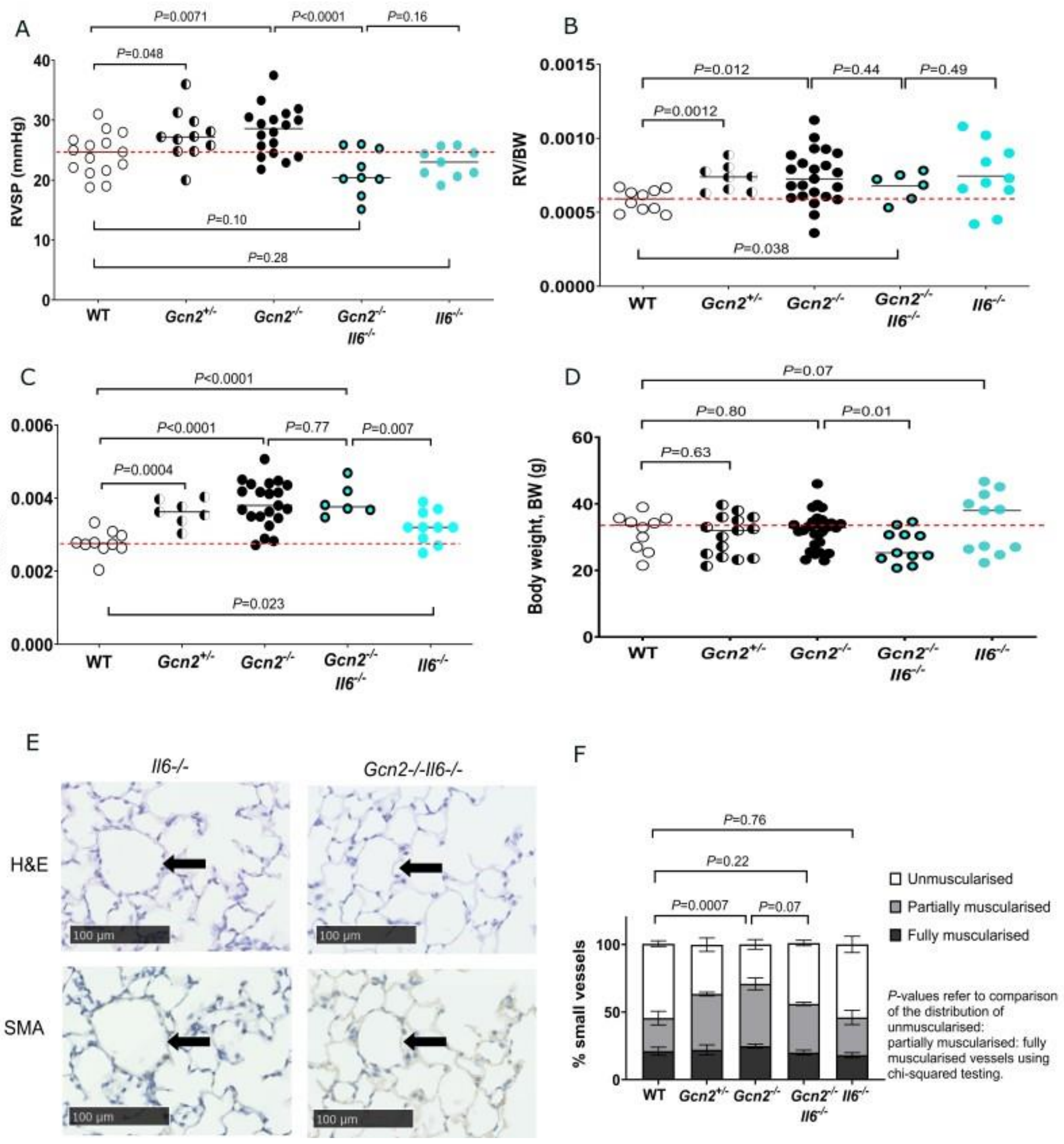

Supplementary Figure 6: Characterisation of the *Il6*-deficient mouse and the *Gcn2*$^{-/-}$*Il6*$^{-/-}$ mouse at baseline.

Panels A-D show the right ventricular systolic pressure (RVSP, panel A), the right ventricular mass indexed to body weight (RV/BW, panel B), the left ventricular mass indexed to body weight (LV+S/BW, panel C) and the body weight (panel D) in *Il6*$^{-/-}$ and *Gcn2*$^{-/-}$*Il6*$^{-/-}$ mice. Note that this panel includes mice whose data has been previously shown in Figure 1 (for wild-type, *Gcn2*$^{+/-}$ and *Gcn2*$^{-/-}$ mice) as by UK Home Office regulations we are required to use the

minimum number of mice to fulfil experimental requirements. Panels E-F show representative histological sections of the lungs of *Il6*$^{-/-}$ and *Gcn2*$^{-/-}$*Il6*$^{-/-}$ mice, stained with haematoxylin and eosin (H&E) and smooth muscle actin (SMA) and the quantification of non-muscularised, partially muscularised and fully muscularised vessels (panel F). In panels (A-D) the median is shown, and comparisons have been made using unpaired t-tests (for parametric data) or Mann-Whitney tests (for non-parametric data). In panel F the distribution of non-muscularised: partially muscularised: fully muscularised vessels has been compared between groups using chi-squared testing.

References

1. Raczy, C., et al., *Isaac: ultra-fast whole-genome secondary analysis on Illumina sequencing platforms.* Bioinformatics, 2013. **29**(16): p. 2041-3.
2. Li, H., *A statistical framework for SNP calling, mutation discovery, association mapping and population genetical parameter estimation from sequencing data.* Bioinformatics, 2011. **27**(21): p. 2987-93.
3. Evans, C.E., et al., *Endothelial cells in the pathogenesis of pulmonary arterial hypertension.* Eur Respir J, 2021. **58**(3).
4. Lechartier, B., et al., *Phenotypic Diversity of Vascular Smooth Muscle Cells in Pulmonary Arterial Hypertension: Implications for Therapy.* Chest, 2022. **161**(1): p. 219-231.
